# Characterizing spatial gene expression heterogeneity in spatially resolved single-cell transcriptomic data with nonuniform cellular densities

**DOI:** 10.1101/gr.271288.120

**Published:** 2021-10

**Authors:** Brendan F. Miller, Dhananjay Bambah-Mukku, Catherine Dulac, Xiaowei Zhuang, Jean Fan

**Affiliations:** 1 Department of Biomedical Engineering, Johns Hopkins University, Baltimore, Maryland 21218, USA;; 2 Center for Computational Biology, Johns Hopkins University, Baltimore, Maryland 21211, USA;; 3 Howard Hughes Medical Institute, Cambridge, Massachusetts 02138, USA;; 4 Department of Molecular and Cellular Biology, Cambridge, Massachusetts 02138, USA;; 5 Department of Chemistry and Chemical Biology, Cambridge, Massachusetts 02138, USA;; 6 Department of Physics, Harvard University, Cambridge, Massachusetts 02138, USA

## Abstract

Recent technological advances have enabled spatially resolved measurements of expression profiles for hundreds to thousands of genes in fixed tissues at single-cell resolution. However, scalable computational analysis methods able to take into consideration the inherent 3D spatial organization of cell types and nonuniform cellular densities within tissues are still lacking. To address this, we developed MERINGUE, a computational framework based on spatial autocorrelation and cross-correlation analysis to identify genes with spatially heterogeneous expression patterns, infer putative cell–cell communication, and perform spatially informed cell clustering in 2D and 3D in a density-agnostic manner using spatially resolved transcriptomic data. We applied MERINGUE to a variety of spatially resolved transcriptomic data sets including multiplexed error-robust fluorescence in situ hybridization (MERFISH), spatial transcriptomics, Slide-seq, and aligned in situ hybridization (ISH) data. We anticipate that such statistical analysis of spatially resolved transcriptomic data will facilitate our understanding of the interplay between cell state and spatial organization in tissue development and disease.

Characterization of the spatial context of cells and their cellular states is essential to understanding the connection between tissue organization and function, particularly in complex organs such as the mammalian brain. Furthermore, spatial context plays an important role in development and organ formation in multicellular organisms, as well as in aberrant processes such as cancer (Crosetto et al. 2015). Although advances in single-cell sequencing technologies can be used to discover transcriptionally distinct subpopulations of cells in an unbiased manner, current protocols require dissociating cells from tissue, thereby losing valuable spatial context (Crosetto et al. 2015). Thus, how these subpopulations of cells are organized in space and how they may interact with each other remains an open question in many systems.

To preserve informative spatial context, recent advances in imaging-based approaches have enabled in situ spatially resolved transcriptomic profiling with single-cell resolution (Zhuang 2021). In addition, approaches based on spatially resolved RNA capture followed by sequencing, such as spatial transcriptomics and Slide-seq, provide spatially resolved untargeted transcriptomic profiling at the pixel level, with pixel size of 10–100 µm (Larsson et al. 2021). Such high throughput data generation, both in terms of the number of genes and number of cells assayed, demands scalable computational methods that take advantage of this new spatial dimension to efficiently identify statistically significant spatial patterns and relationships. In addition, as these methods are applied to increasingly complex tissues, statistical analyses must be able to accommodate the nonuniform cell density induced by biological factors, such as the presence of multiple, often spatially organized, cell types inherent to tissues, as well as technical factors, such as distortions from tissue sectioning.

Three statistical methods, SpatialDE, Trendsceek, and SPARK have previously been developed to identify spatial gene expression heterogeneity, defined as an uneven, aggregated, or patterned spatial distribution of gene expression magnitudes (Edsgärd et al. 2018; Svensson et al. 2018; Sun et al. 2020). Briefly, SpatialDE identifies spatial gene expression heterogeneity by decomposing a gene's expression variance into a spatial and a nonspatial component using a spatial variance term that incorporates the pairwise distances between cells. Trendsceek characterizes spatial gene expression heterogeneity by testing a gene's expression for dependence with the pairwise distances between cells. SPARK identifies spatial gene expression heterogeneity that best fits the observed gene expression trends using multiple linear spatial models based on different Gaussian and periodic kernel functions that incorporate distances between cells. Thus, each method directly incorporates information regarding cell distances, which could present a challenge for analyses within tissues where cells are distributed with nonuniform densities. For example, where local cell density is higher and the distance between cells are smaller, randomly varying gene expression may give rise to apparent spatial aggregation owing to cellular aggregation (Supplemental Fig. S1A). Likewise, spatial variation in cellular density could also potentially mask spatial variation in gene expression (Supplemental Fig. S1B). It is, however, important to identify variations in gene expression magnitudes across cells that do not arise from variations in cellular density. Alternative approaches such as SpaOTsc can accommodate nonuniform cellular densities if provided with geodesic distances (Cang and Nie 2020). Briefly, using such density-agnostic geodesic distances relating cells in space, SpaOTsc uses optimal transport to estimate how much information about each gene's expression magnitude can be provided by another gene's expression magnitude to identify groups of genes with similar spatial patterning. However, this approach does not provide a statistical framework to distinguish between significantly spatially heterogeneous genes versus nonsignificant or nonspatially heterogeneous genes. Furthermore, cells in tissues inherently exist in a three-dimensional context, yet computational approaches capable of taking into consideration *z*-axis information, often at differing length scales such as multiple noncontiguous tissue sections, have yet to be shown. Here, we developed MERINGUE, a density-agnostic method for identifying spatial gene expression heterogeneity using spatial autocorrelation and cross-correlation analyses. Using a variety of spatially resolved transcriptomic data sets, we show that MERINGUE is able to identify biologically relevant spatial gene expression patterns in both 2D and 3D in a manner that is independent of cell density.

## Results

### Overview of MERINGUE

**Figure 1.**
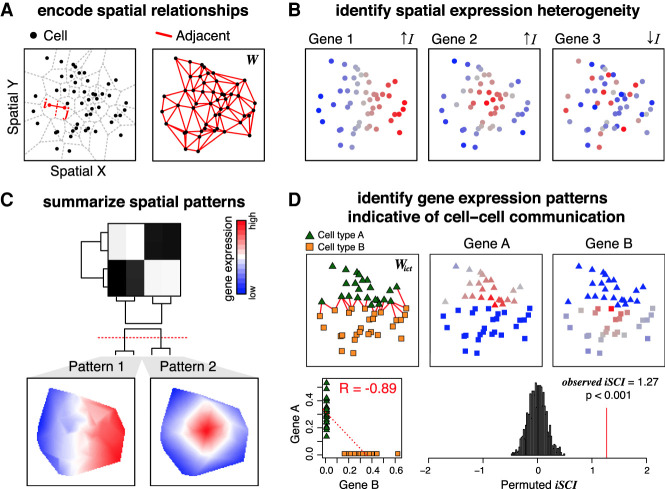
Overview of MERINGUE. (*A*) MERINGUE encodes spatial relationships among spatially resolved data sets, such as cells, using a binary adjacency weight matrix *W*. (*Left*) Two cells are considered adjacent if their neighborhoods inferred from Voronoi tessellation share an edge. (*Right*) The binary adjacency weight matrix *W* is visualized by plotting all cells in space with a red line connecting cells if cells are adjacent and no line otherwise. (*B*) MERINGUE identifies genes with spatially heterogeneous expression using *W* to compute and evaluate the significance of a spatial autocorrelation index *I* for each gene. When a gene's expression magnitude between spatially adjacent cells are highly correlated, *I* will be positive, indicative of spatial heterogeneity. Three simulated gene expression profiles are provided to illustrate examples of high and low spatial heterogeneity with red indicating high expression and blue indicating low expression. (*C*) MERINGUE groups identified spatially heterogeneous genes into primary spatial patterns by computing a spatial cross-correlation index for every gene pair. (*Top*) The resulting spatial cross-correlation matrix is used to construct a hierarchical dendrogram. (*Bottom*) Dynamic tree cutting is applied to partition genes into patterns. Groups of genes are *Z*-scored and averaged, with empty regions filled in using Akima interpolation to visualize final patterns. (*D*) MERINGUE identifies gene expression patterns that may be indicative of putative cell–cell communication using an inter-cell-type weight matrix *W*_*ict*_ between two cell types, which can then be used to compute an inter-cell-type spatial cross-correlation index *i*SCI between two genes (*Top left*). Two cell types, A and B, are shown as green triangles and orange squares, respectively. *W*_*ict*_ is visualized with a red line for cells of cell type A spatially adjacent to cells of cell type B. (*Top right*) Cell type–A cells express gene A at variable levels, whereas cell type–B cells express gene B at variable levels, with red indicating high expression and blue indicating low expression. (*Bottom left*) Cell type–A cells do not express gene B and cell type–B cells do not express gene A, resulting in a generally negative Pearson's correlation (*R*) between the two genes. (*Bottom right*) However, expression of gene A in cells of cell type A is highly correlated with the expression of gene B in spatially adjacent cells of cell type B, resulting in a positive *i*SCI. The significance of this *i*SCI is assessed by permutation.

Given a set of spatial positions such as those corresponding to single cells, MERINGUE first represents these cells as neighborhoods using Voronoi tessellation (Fig. 1A). In Voronoi tessellation, planes are partitioned into neighborhoods where a neighborhood for a cell consists of all points closer to that cell than any other (Okabe et al. 1992). Cells are then considered adjacent if their neighborhoods share an edge. For biological interpretability, we further require adjacent cells to be within a certain spatial distance in space to accommodate realistic length scales of cellular interactions. This neighborhood representation of cells accommodates varying neighborhood sizes and distances between cells and thus can characterize cell types and tissues with nonuniform densities. We also find that such neighborhood adjacency relationships to be more stable than *k*-nearest-neighbor or *k*-mutual-nearest-neighbor relationships, because such relationships require *k* to be specified beforehand and a single *k* value may not be appropriate for all densities and regions within a spatially resolved data set (Supplemental Fig. S2A). MERINGUE encodes these adjacency relationships using a binary adjacency weight matrix *W* with a weight of 1 if two data sets are adjacent and 0 otherwise (Fig. 1A). Such adjacency relationships are not restricted to 2D and thus can accommodate 3D information, such as from imaging of multiple slices of the same tissue or 3D volumetric imaging of a tissue block, if available (Lee et al. 2015; Wang et al. 2018).

Next, to identify genes with spatially heterogeneous expression, given a matrix of normalized gene expression magnitudes for the same set of spatially resolved cells, MERINGUE uses this adjacency weight matrix *W* in calculating Moran's *I*, a global measure of spatial autocorrelation popular in geospatial analysis, for each gene's expression magnitude (*x*) across the population of *N* cells (Moran 1950):

$${\mathrm{Moran}}^{\prime }s\;I=\frac{N}{\sum_{i}^{N}\sum_{j}^{N}W_{ij}}\frac{\sum_{i}^{N}\sum_{j}^{N}W_{ij}(x_{i}-\bar{x})(x_{j}-\bar{x})}{\sum_{i}^{N}{\left(x_{i}-\bar{x}\right)}^{2}}.$$

When a gene's expression magnitude (*x*) between spatially adjacent cells (*W*_*ij*_ = 1) are positively correlated, Moran's *I* will be positive (Fig. 1B), indicative of spatial gene expression heterogeneity. Moran's *I* has a closed form, allowing *P*-values to be derived without reliance on computationally intensive permutations (Supplemental Fig. S2B; Moran 1950).

To further characterize the scale of significant spatial gene expression heterogeneity, using the same matrix of normalized gene expression magnitudes and adjacency weight matrix *W*, MERINGUE calculates a local indicator of spatial association (LISA) for each gene (Anselin 1995):

$${\mathrm{LISA}}_{i}=N\frac{\left(x_{i}-\bar{x})\sum_{j}^{N}W_{ij}(x_{j}-\bar{x}\right)}{\sum_{i}^{N}{\left(x_{i}-\bar{x}\right)}^{2}}.$$

When a gene's expression values (*x*) in a given cell (*i*) is positively correlated with that cell's spatially adjacent neighbors, the cell's LISA for the given gene will be highly positive. Again, LISA has a closed form, allowing *P*-values to be derived quickly. As such, MERINGUE defines the percent of cells with statistically significant LISAs as the percent of cells driving a spatially heterogeneous gene expression pattern. This use of LISA guards against the identification of spatially heterogeneous genes driven by small hotspots or outliers. Simulations suggest that false positives may be effectively eliminated by restricting to spatial heterogeneity driven by >5% of cells (Supplemental Fig. S2C).

Finally, to summarize genes into primary spatial patterns, MERINGUE calculates a spatial cross-correlation index between all pairs of genes identified with significant spatially heterogeneous expression driven by a sufficient percentage of cells:

$$\mathrm{SCI}=\frac{N}{2\sum_{i}^{N}\sum_{j}^{N}W_{ij}}\frac{\sum_{i}^{N}\sum_{j}^{N}W_{ij}(x_{i}-\bar{x})(y_{j}-\bar{y})}{\sqrt{\sum_{i}^{N}{\left(x_{i}-\bar{x}\right)}^{2}}\sqrt{\sum_{j}^{N}{\left(y_{j}-\bar{y}\right)}^{2}}}.$$

When one gene's expression magnitude (*x*) in a given cell (*i*) is positively correlated with another gene's expression magnitude (*y*) in the cell's spatially adjacent neighbors (*j*), the SCI for this gene pair will be positive. MERINGUE computes this spatial cross-correlation index for all gene pairs to derive a spatial cross-correlation matrix that is then used for hierarchical clustering and dynamic tree cutting to group these genes into primary spatial patterns (Fig. 1C; Langfelder et al. 2008).

In addition, MERINGUE further builds on this spatial cross-correlation index to identify spatially cross-correlated gene expression patterns that may be indicative of cell–cell communication. In particular, communicating cell types may express higher levels of particular ligand genes while being spatially adjacent to cells that express higher levels of corresponding receptor genes or vice versa. Thus, to identify such gene expression patterns that may be indicative of putative cell–cell communication, MERINGUE constructs an adjacency weight matrix *W* to only include adjacency relationships between cell types and calculates the spatial cross-correlation statistics for known receptor and ligand genes (Ramilowski et al. 2015). In this manner, when a receptor gene's expression magnitude (*x*) in a given cell (*i*) of cell type A is positively correlated with the corresponding ligand gene's expression magnitude (*y*) in cells of cell type B among the cell's spatially adjacent neighbors (*j*), the inter-cell-type SCI for this cell type pair will be highly positive. Statistical significance can then be assessed by permutation testing (Fig. 1D).

### MERINGUE identifies genes with spatially heterogeneous expression patterns and is robust to changes in cellular densities

**Figure 2.**
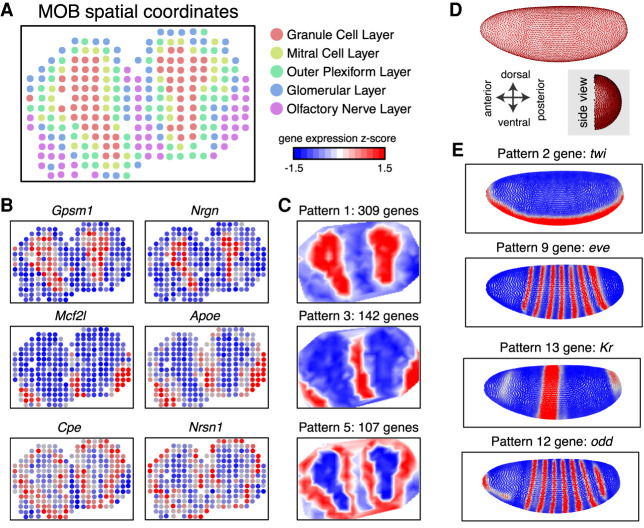
Application of MERINGUE to 2D spatial transcriptomic data of the main olfactory bulb (MOB) and 3D-aligned ISH data of the *Drosophila melanogaster* embryo. (*A*) Spatially unaware single-cell clustering analysis identifies five transcriptionally distinct clusters corresponding to various known cell layers in the MOB. Spatial spots are colored based on their inferred cell layer annotation. (*B*) MERINGUE identifies genes with significantly spatially heterogeneous expression in the MOB. Select genes are shown. (*C*) MERINGUE groups genes with significantly spatially heterogenous expression in the MOB into five primary spatial patterns. Select patterns are shown. (*D*) MERINGUE's adjacency weight matrix visualized for aligned 3D in situ hybridization data of the *D. melanogaster* embryo. Each point is an aligned cell. Cells are connected with a red line if they are inferred to be adjacent. A top view and rotated side view are shown. (*E*) MERINGUE groups genes into spatial patterns in the *D. melanogaster* embryo. Representative genes from select identified patterns are shown.

As a proof of principle, we first applied MERINGUE to spatial transcriptomics (ST) data of the mouse main olfactory bulb (MOB) and Slide-seq data of the mouse cerebellum (Ståhl et al. 2016; Rodriques et al. 2019). Briefly, for ST and Slide-seq, RNAs from tissue sections are captured onto an array of DNA barcoded spots or a monolayer of DNA barcoded beads, respectively. By resolving the DNA barcodes, both approaches enable matching of detected RNA abundances with their original spatially resolved spots or beads, resulting in RNA sequencing measurements with uniformly gridded 2D positional information. To validate MERINGUE, we expected that identified spatially heterogeneous genes in the MOB should mark transcriptionally distinct and spatially organized cell layers or combinations of cell layers (Fig. 2A; Supplemental Fig. S3A). Indeed, when we applied MERINGUE to analyze 7365 genes among 260 spots, of the 834 identified as significantly spatially heterogeneous genes (adjusted *P*-value < 0.05) driven by >5% of spots (Fig. 2B; Supplemental Fig. S3B; Supplemental Table S1), 90% (754/834) overlapped with genes that are significantly differentially expressed genes across cell layers (adjusted *P*-value < 0.05) as identified from ANOVA testing. Furthermore, these 834 spatially heterogeneous genes can be further partitioned into five primary spatial patterns that correspond to cell layers and combinations of cell layers as expected (Fig. 2C; Supplemental Fig. S3C). One well-characterized aspect of spatial organization in the MOB involves the convergence of axonal projections from olfactory receptor neurons expressing a given olfactory receptor (*Olfr*) into glomerular neuropils at fixed locations in the glomerular cell layer of the olfactory bulb (Ressler et al. 1994; Vassar et al. 1994; Mombaerts et al. 1996). Therefore, as an additional validation, we evaluated whether *Olfr* genes were spatially heterogeneous in a pattern that corresponds to the glomerular and surrounding cell layers. Although individual *Olfr* genes are very lowly expressed such that detection was generally limited to only a few copies in a few spots (Supplemental Fig. S3D), rendering assessment of spatial heterogeneity for individual *Olfr* genes infeasible, by aggregating the expression of all detected *Olfr* genes, we validate that MERINGUE was able to identify significant spatial heterogeneity (*P*-value = 0.0000283). The spatial expression pattern further corresponded approximately to the glomerular and surrounding cell layer as expected (Supplemental Fig. S3E). For Slide-seq data of the mouse cerebellum, we applied MERINGUE to analyze 9762 genes among 1589 beads previously annotated to correspond to the Purkinje layer (Supplemental Fig. S4A). We validate that *Aldoc* (also known as zebrin II) is identified as among the most significantly spatially heterogeneous genes (adjusted *P*-value < 0.05, >5% beads) (Supplemental Table S2), consistent with observations from the original publications (Rodriques et al. 2019).

We next compared MERINGUE to previously published computational methods for analyzing spatially resolved transcriptomic data, SpatialDE and SPARK (Svensson et al. 2018; Sun et al. 2020). We applied each method to analyze 7365 genes among 260 spots in the MOB to identify spatially heterogeneous genes (Supplemental Methods). We found the resulting significance of spatial heterogeneity in terms of −log_10_(adjusted *P*-value) to be highly correlated across genes between all tested computational methods (R = 0.914 between MERINGUE and SpatialDE, *R* = 0.898 between MERINGUE and SPARK) (Supplemental Fig. S5A,B). The resulting set of significantly spatially heterogeneous genes identified by each tested computational method using a common significance threshold (adjusted *P*-value < 0.05) were also highly overlapping (Supplemental Fig. S5C). We further evaluated the computational efficiency of each method in terms of runtime and memory usage as a function of the number of genes and the number of cells in the data set (Supplemental Methods). We found that MERINGUE achieves improved computational efficiency compared to previously published computational methods (Supplemental Fig. S5D,E). Thus, MERINGUE is capable of identifying spatially heterogeneous genes consistent with previously published approaches in a scalable manner.

We developed MERINGUE to accommodate the nonuniform cellular densities inherent to tissues. Thus, we reasoned that changes in cellular densities should not substantially impact MERINGUE's ability to identify spatially heterogeneous genes. To assess MERINGUE's robustness to spatial variations in cellular densities, we artificially induced nonuniformity in the spatial distribution of ST spots by distorting their positional coordinates (Supplemental Methods; Supplemental Fig. S6A). Owing to its use of a distance-agnostic binary weight matrix, MERINGUE's resulting significance of spatial heterogeneity across genes was highly correlated between the uniform and nonuniform case as expected (Spearman's ρ = 0.862) (Supplemental Fig. S6B). Likewise, although MERINGUE was able to identify 834 significantly spatially heterogeneous genes (adjusted *P*-value < 0.05, >5% of spots) in the uniform density case, 544 (65%) of these genes were recovered in the nonuniform density case with the same adjusted *P*-value and spot percentage thresholds. The discrepancies between the uniform and nonuniform cases can be largely attributed to changes in the binary weight matrix (Supplemental Fig. S6C). Because SpatialDE and SPARK incorporate Euclidean distances between cells in their evaluation of spatial patterns, we reasoned that spatial variations in cellular density would impact their ability to identify spatially heterogeneous genes. We thus applied the same uniform and artificially induced nonuniform case comparison. As expected, the resulting significance of spatial heterogeneity across genes was less well correlated between the uniform and nonuniform density case for both SpatialDE (Spearman's ρ = 0.427) and SPARK (Spearman's ρ = 0.418) (Supplemental Fig. S6D). Likewise, although SpatialDE was able to identify 360 significantly (adjusted *P*-value < 0.05) spatially heterogeneous genes in the uniform density case, only 56 (16%) of these genes were recovered in the nonuniform case with the same adjusted *P*-value threshold. Similarly, SPARK was able to identify 664 significantly (adjusted combined *P*-value < 0.05) spatially heterogeneous genes in the uniform case, but only 66 (10%) of these genes were recovered in the nonuniform case with the same adjusted *P*-value threshold.

### MERINGUE integrates 3D and multilayer tissue information

Although spatially resolved transcriptomics measurements generally provide positional information in the imaging (*x*–*y*) plane, *z*-direction information can be obtained through optically scanning through imaging planes or sequential tissue sections. To show integration of *z*-direction information, we first applied MERINGUE to 3D in situ hybridization (ISH) data, aligned across multiple stage 6 *Drosophila melanogaster* embryos for 84 selected marker genes (Fig. 2D; Supplemental Methods; Fowlkes et al. 2008; Karaiskos et al. 2017). The role of spatial patterning in shaping cellular identities has been well established in the *D. melanogaster* embryo and as such, the 84 marker genes were previously chosen for their known spatial patterning. Indeed, we validated that all 84 genes are identified by MERINGUE as significantly spatially heterogeneous (adjusted *P*-value < 0.05, >5% spots) as expected. We further validated that these genes could be grouped by MERINGUE into 14 primary spatial patterns that correspond to known regionally confined developmental fates and layers of the segmentation gene network (Fig. 2E; Supplemental Fig. S7; Supplemental Table S3; Ingham 1988; Karaiskos et al. 2017). For example, pattern 2 corresponds to the mesoderm and includes mesoderm determinant gene *twist* (*twi*), whereas pattern 13 corresponds to the thoracic segments and includes known gap gene *Kruppel* (*Kr*) (Preiss et al. 1985; Leptin 1991). Similarly, patterns 9 and 12 correspond to two spatially alternating striped patterns that include known pair-rule genes *even skipped* (*eve*) and *odd skipped* (*odd*), respectively (Macdonald et al. 1986; Coulter et al. 1990).

Alternatively, z-information may be derived through serial sections. We thus next applied MERINGUE to spatial transcriptomic data of four consecutive histological sections of a human breast cancer biopsy (Supplemental Methods; Ståhl et al. 2016). Analyzing each section independently, we identified 414 genes that show significant spatial variability (adjusted *P*-value < 0.05, >5% spots) in at least one section out of 6214 genes tested (Supplemental Table S4). Because the distance between cells across serial sections are greater than the distances between cells within sections, we sought to identify spatial patterns consistent across layers by testing for spatial correlation between mutual nearest neighbors in space across sections (Supplemental Fig. S8A). Such a multilayer integrated approach confirmed 242 significantly spatially heterogeneous genes as being consistent across sections (Supplemental Table S4; Supplemental Fig. S8B). Of the remaining 172 genes that were identified as significantly spatially heterogeneous in individual sections but not across sections, visual inspection showed that although these genes show spatial variability within sections, there was minimal correspondence across sections (Supplemental Fig. S8C). Such transcriptional patterns may be indicative of layer-specific subpopulations or transcriptional features. For structurally stereotypic tissues, consistency across tissue sections may be used as an additional criterion for identifying functionally relevant spatial patterns. Thus, MERINGUE is capable of accommodating 3D information to identify spatially heterogeneous genes in 3D as well as genes with spatial expression patterns consistent across serial sections.

### MERINGUE identifies spatial patterns in the mouse hypothalamic preoptic region using spatially resolved single-cell gene expression data by MERFISH

Particularly in complex organs such as the mammalian brain, the ability to identify and interrogate the spatial organization of cell types may provide additional insights into potential functional roles underlying the spatial organization of neuronal populations (y Cajal 1911; Amaral and Witter 1989; Arber 2012). We applied MERINGUE to analyze spatially resolved single-cell transcriptomic data of the hypothalamic preoptic region obtained using multiplexed error-robust fluorescence in situ hybridization (MERFISH) (Moffitt et al. 2018). Briefly, MERFISH allows individual RNA molecules in cells to be imaged and identified by using a combinatorial labeling strategy that encodes RNA species with error-robust barcodes that can be read out bit-by-bit using sequential rounds of single-molecule fluorescence in situ hybridization (Chen et al. 2015). MERFISH has enabled simultaneous detection and identification of thousands of targeted RNA species, which can then be segmented into cells to provide spatially resolved single-cell transcriptome measurements (Chen et al. 2015; Xia et al. 2019). Moffitt et al. (2018) previously used a 155 gene panel to characterize the hypothalamic preoptic region (1.8 × 1.8 × 0.6 mm, Bregma +0.26 to −0.34) in adult mice to identify 31 excitatory and 39 inhibitory neuronal subtypes in addition to non-neuronal cell types using graph-based community-detection clustering analysis that relies solely on the gene expression of profiles of cells without considering the spatial information.

**Figure 3.**
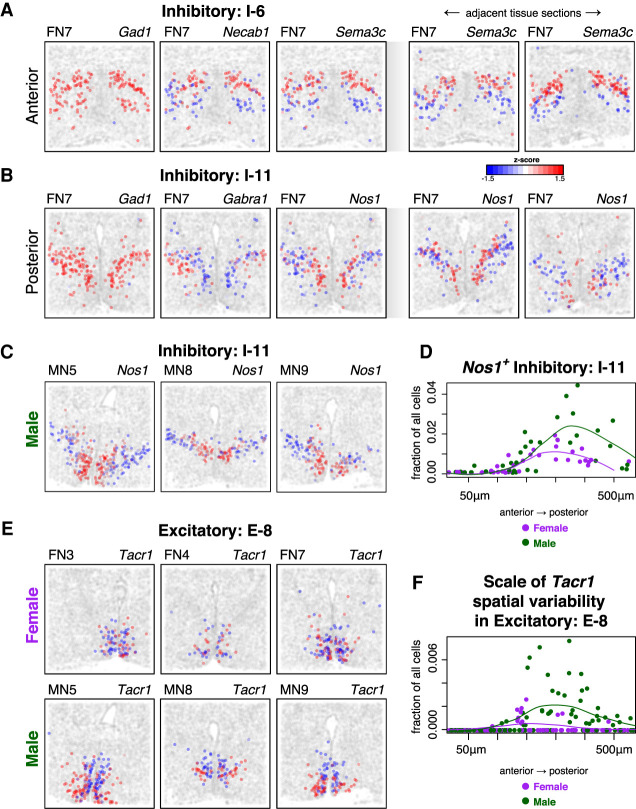
MERINGUE identifies spatial heterogeneity within cell types in the preoptic region of the mouse hypothalamus using MERFISH. (*A*, *left*) Expression of three sample genes in Inhibitory I-6 neurons in female naïve animal 7 (FN7). Each point is a cell. Cells are colored by expression with red denoting high expression and blue denoting low expression. Cells that are not I-6 cells are colored in gray. *Gad1* is highly expressed in all I-6 neurons, whereas *Necab1* and *Sema3c*show significant spatial variation. (*Right*) Expression of *Sema3c* in I-6 in adjacent tissue sections in FN7 show similar spatial patterning. (*B*, *left*) Expression of three sample genes in Inhibitory I-11 neurons in FN7. Again, *Gad1* is highly expressed in all I-11 cells, but *Gabra1* and *Nos1* show significant spatial variation. (*Right*) Expression of *Nos1* in I-11 neurons in adjacent tissue sections in FN7 show similar spatial patterning. (*C*) Expression of *Nos1* in I-11 neurons in representative male naïve animals MN5, MN8, and MN9 show similar spatial patterns to the female animal in *B*. (*D*) Fraction of *Nos1*^+^I-11 cells for male and female animals across tissue layers from the anterior to posterior preoptic region. Each dot represents one tissue layer in one animal. Lines represent fitted curves for males and female animals. (*E*) Expression of *Tacr1* in E-8 neurons in female (*top*) and male (*bottom*) animals. (*F*) Scale spatial heterogeneity of *Tacr1* in E-8 neurons for male and female animals across tissue layers from the anterior to posterior preoptic region. Each dot represents one tissue layer in one animal. Lines represent fitted curves for male and female animals.

We applied MERINGUE to analyze the 155 genes along with five blank control barcodes, DAPI, and poly(dT) signals as negative controls within each cell type and subtype to identify additional aspects of spatial heterogeneity. Applying a rigorous approach to identify genes with spatially heterogeneous expression patterns that are consistent across tissue layers and reproducible across animals (Supplemental Methods), we were able to identify at least one such spatially heterogeneous gene in 34 of 83 cell types and subtypes analyzed (Fig. 3A; Supplemental Fig. S9; Supplemental Table S5). None of the blank control barcodes, DAPI, or poly(dT) signals were identified as consistently spatially variable. MERINGUE further identified significant spatial gene expression heterogeneity within neuronal subtypes in both the anterior and posterior of the preoptic region. Likewise, spatial gene expression heterogeneity was identified in both inhibitory and excitatory neuronal subtypes. These aspects of spatial heterogeneity were consistent with previous published spatially unaware variance and principal components-based analyses and visual inspection (Moffitt et al. 2018).

By providing a quantitative framework to systematically identify and evaluate the statistical significance of spatial gene expression heterogeneity, MERINGUE identified that cells of inhibitory subtype I-6 in the anterior of the preoptic region can be partitioned into a superior and inferior spatial lobe marked by higher and lower expression of *Sema3c* and *Necab1*, respectively (Fig. 3A). These patterns are consistent across adjacent tissue sections. Likewise, cells of inhibitory subtype I-11 in the posterior preoptic region can be partitioned into a medial and lateral spatial group marked by lower expression of *Gabra1*, higher expression of *Nos1*, and higher expression of *Gabra1* lower expression of *Nos1,* respectively, and this partition is consistent across adjacent tissue sections (Fig. 3B). Alternatively, *Gad1*, which marks inhibitory cells, is highly expressed among all cells and does not show significant spatial heterogeneity as expected. Inhibitory subtype I-11 was previously identified to be specifically activated by male mating based on the expression of immediate early gene *Fos* (Moffitt et al. 2018). Although inhibitory subtype I-11 showed significant spatial heterogeneity in both male and female animals (Fig. 3C), we found the fraction of *Nos1*^+^ I-11 neurons to be significantly higher in males than females (Student's *t*-test *P*-value = 0.03656) (Fig. 3D). *Esr1* and *Irs4* were also identified to be significantly spatially heterogeneous in I-11 neurons in a pattern similar to *Nos1* (Supplemental Fig. S9). *Esr1* and *Irs4* have been previously shown to display sex-differences in their expression (Xu et al. 2012; Moffitt et al. 2018). These observations suggest the potential presence of a finer *Nos1*^+^ I-11 neuronal subpopulation that is sexually dimorphic. Furthermore, MERINGUE generally identified concordant spatial gene expression heterogeneity in both male and female animals (Supplemental Fig. S10), but tachykinin receptor 1 (*Tacr1*, also known as neurokinin receptor 1) was identified as significantly spatially heterogeneous in excitatory subtype E-8 neurons only in male and not female mice (Fig. 3E). No other tested neuronal subtype was identified to show such consistently statistically significant sexually dimorphic spatial heterogeneity. Previously, E-8 neurons were identified to be activated in male mice during mating based on expression of *Fos* (Moffitt et al. 2018). However, E-8 neurons did not show a significant difference in terms of their proportion to all cells between female and male mice (Student's *t*-test *P*-value = 0.268). Likewise, we confirmed that the fraction of cells expressing *Tacr1* in E-8 neurons is not significantly different between male and female mice (Student's *t*-test *P*-value = 0.429). However, when we quantified the fraction of cells driving the spatial heterogeneity of *Tacr1* expression based on LISA, we observe a significant difference between male and female mice (Fig. 3F) (Student's *t*-test *P*-value = 0.01316). *Tacr1* knockout mice have been previously observed to show deficits in sexual behavior (Berger et al. 2012). The sexually dimorphic spatial organization of *Tacr1* expression in E-8 neurons may thus suggest a sexually dimorphic difference in connectivity responsible for its sexually dimorphic activation in sexual behavior. In this manner, MERINGUE enables quantitative and systematic evaluation of spatial gene expression heterogeneity within transcriptionally distinct cell subtypes from single-cell spatially resolved transcriptomics data.

### Spatially informed clustering identifies transcriptionally and spatially distinct subtypes of cells

**Figure 4.**
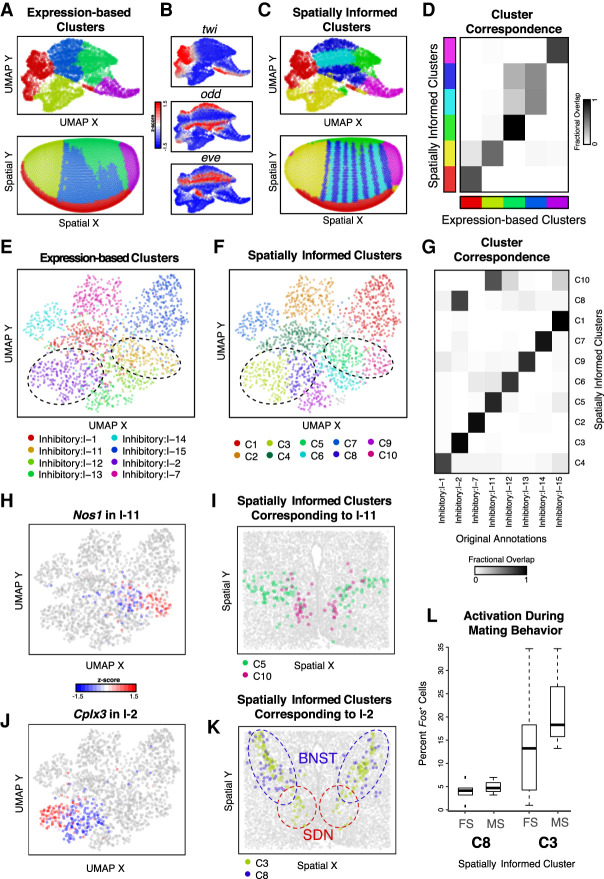
Spatially informed clustering distinguishes spatially distinct subpopulations of cells. (*A*) Expression-based clustering of 3035 stage 6 *D. melanogaster* embryo cells with 84 marker genes by aligned ISH identifies approximately five transcriptionally distinct clusters. (*Top*) UMAP embedding colored by identified cluster annotations. (*Bottom*) Spatial coordinates colored by identified cluster annotations. (*B*) Expression of select marker genes on the UMAP embedding with red denoting high expression and blue denoting low expression. (*C*) Spatially informed clustering splits expression-based clusters in a spatially coherent manner. (*Top*) Again, UMAP embedding colored by identified spatially informed cluster annotations. (*Bottom*) Spatial coordinates colored by identified spatially informed cluster annotations. (*D*) Correspondence between expression-based clusters in *A* and spatially informed clusters in *C* highlights high correspondence between most clusters with the exception of one cluster being split into two. (*E*) UMAP embedding of populous inhibitory neuronal subtypes in one posterior preoptic tissue section from one animal measured using MERFISH, where each point is a cell colored by the original subtype annotations. (*F*) Same UMAP embedding as *E* in which each point is a cell colored by the spatially informed clustering annotation. Black dashed lines highlight clusters that have now split. (*G*) Correspondence between expression-based clusters in *E* and spatially informed clusters in *F* highlights high correspondence between most clusters with the exception of cells originally annotated as I-2 and I-11 now being split into two. (*H*) Same UMAP embedding as *E* in which each point is a cell colored by *Nos1*expression for cells originally annotated as I-11. (*I*) Spatial location of cells within the tissue colored by their spatially informed cluster assignment for cells originally annotated as I-11. (*J*) Same UMAP embedding as *E*, in which each point is a cell colored by *Cplx3* expression for cells originally annotated as I-2. (*K*) Spatial location of cells within the tissue colored by their spatially informed cluster assignment for cells originally annotated as I-2. Regions corresponding to the BNST and SDN are highlighted with blue and red dashed lines, respectively. Representative slice in representative animal shown. (*L*) Percentage of activated cells based on *Fos* expression during female (FS) and male (MS) sexual behavior for spatially informed clusters C3 and C8 originally annotated as I-2. Boxes in the box plot denote the median values and inner quartile ranges (IQR), and whiskers denote 1.5 × IQR with additional outliers represented as points.

Spatial organization may play an important role in shaping cellular identities. Likewise, we may expect unsupervised clustering based on transcriptional profiles alone to recover spatially organized cell populations. However, for the aligned ISH data of the *D. melanogaster* embryo, we find such gene expression clustering analysis to aggregate cells expressing different pair-rule genes into a single transcriptional cluster owing to these cells sharing many other commonly up-regulated and down-regulated genes despite their spatially distinct organization (Fig. 4A,B), consistent with previously published analyses (Karaiskos et al. 2017). However, as our spatial analysis was able to distinguish between the two alternating striped spatial patterns marked by expression of pair-rule genes *eve* and *odd*, respectively (Fig. 2E), we sought to incorporate spatial information to help distinguish these spatially distinct but transcriptionally similar groups of cells.

Briefly, as in expression-based clustering, we constructed a neighbor graph in which nodes are cells and nodes are connected with an edge if the represented cells that are within the *k*-most transcriptionally similar cells for some user-selected resolution parameter *k*. We incorporated spatial information by weighing the edges of the network by the distance (*d*) between two neighborhoods (*i*, *j*) in the adjacency representation *W* (Methods): (1/*d*_*ij*_ + 1) + 1. Again, use of such a neighborhood representation can accommodate the nonhomogenous density of cells in tissues compared to a Euclidean distance-based measure of spatial distance. In this manner, if two cells are closer in space (*d*_*ij*_ is small), their transcriptional similarity will give greater weight in the graph-based clustering. Incorporating these spatial weights into our graph-based clustering with all other parameters kept constant, we were able to split the cluster of cells expressing either *eve* or *odd* into two subpopulations, as desired (Fig. 4C). Moreover, such spatially informed clustering generally preserved all other subpopulations and did not result in additional splitting for other subpopulations (Fig. 4D). Furthermore, we showed using simulated data how such incorporation of spatial information can be used to distinguish transcriptionally identical but spatially distinct clusters of cells (Supplemental Fig. S11). In a biological setting, however, cells from the same cell type may populate spatially distinct locations, but such distinct spatial locations alone would not necessarily indicate the presence of finer subtypes. We thus suggest that such spatially informed clustering to be complementary to differential expression analysis, whereby identified spatially distinct cell subpopulations should be analyzed for significantly differentially expressed genes to ensure the presence of significant, likely subtle, transcriptional differences. Therefore, by incorporating spatial information, in conjunction with differential expression analysis, we can identify finer, transcriptionally and spatially distinct subpopulations.

Having shown that incorporation of spatial information via graph weighting can be applied to identify finer transcriptionally and spatially distinct subpopulations of cells, we next sought to apply this approach to identify finer neuronal subtypes in the preoptic region profiled by MERFISH (Moffitt et al. 2018). Focusing on inhibitory neurons, we performed spatially informed clustering analysis on all inhibitory cells in the same animal and tissue layer and compared resulting clusters to previous annotations (Fig. 4E,F; Supplemental Methods). We found that among the eight most populous inhibitory neuronal subtypes (clusters with >100 cells each), our spatially informed clustering was able to produce comparable clusters with the exception of I-2 and I-11, which were each split into two subtypes (Fig. 4G). I-11 was split into two subtypes, cluster 10 (C10) and cluster 5 (C5), that significantly differentially expressed genes including *Nos1* (Fig. 4H; Supplemental Fig. S12A), consistent with our observations of significant spatial heterogeneity in *Nos1* expression among I-11 neurons. Indeed, the two I-11 subtypes appear to be spatially distinct with C10 positioned more medially and C5 more laterally in the posterior preoptic region (Fig. 4I). Likewise, I-2 was split into two subtypes, cluster 3 (C3) and cluster 8 (C8), that significantly differentially up-regulated genes including *Cplx3* and *Dgkk*, respectively (Fig. 4J; Supplemental Fig. S12B,C). Previously, I-2 neurons were observed to overlap with both the sexually dimorphic nucleus of the preoptic area (SDN-POA) as well as other anatomical nuclei such as the bed nucleus of the stria terminalis (BNST) (Moffitt et al. 2018). By refining I-2 into two finer subtypes, C8 is observed to overlap more so with the BNST, while C3 comparably more so with the SDN-POA (Fig. 4K; Supplemental Fig. S12C). I-2 neurons were previously observed to show sexually dimorphic activation during mating and aggression based on the expression of immediate early gene *Fos* (Moffitt et al. 2018). When we compare activation of the two I-2 subtypes based on significant *Fos* expression, we observe comparatively greater activation during mating behavior in one subtype than the other (Fig. 4L). This suggests that the activation in I-2 neurons observed previously may be driven by one of the two I-2 subtypes. Although tuning parameters for regular graph-based clustering without spatial information can also achieve splitting of I-2 and I-11, other inhibitory neuronal clusters can become over split (Supplemental Fig. S12D). Therefore, by incorporating spatial information via graph weighting, MERINGUE provides an alternative approach to tease apart spatially distinct subpopulations without impacting other transcriptionally distinct subtypes.

### MERINGUE identifies putative cell–cell communication between cell types

Spatially resolved transcriptomic data offers opportunity to identify gene expression patterns that may be indicative of putative cell–cell communication between spatially colocalized cell types. Previous computational approaches for inferring cell–cell communication from single-cell RNA-sequencing data have relied on correlations or coexpression of receptor genes in one cell type and corresponding expression of ligand genes in another cell type (Ramilowski et al. 2015; Vento-Tormo et al. 2018; Smillie et al. 2019; Fan et al. 2020). Spatially resolved transcriptomic data provide the opportunity to infer potential cell–cell communication by identifying spatially complementary expression patterns between genes corresponding to interacting surface proteins such as receptors and ligands on spatially neighboring cells. To enable such analyses, we further build on MERINGUE's spatial cross-correlation functionalities by developing an inter-cell-type spatial cross-correlation function to identify potential complementary spatial patterns of gene expression across spatially colocalized cell types (Fig. 1D; Supplemental Fig. S13A–D). However, unlike the spatial autocorrelation function, this inter-cell-type spatial cross-correlation function is not solvable; thus significance must be assessed using permutation to derive a null model. We enhance computational efficiency by implementing a parallelized, adaptive permutation testing approach and assess significance using a permutation-based random label null model. We confirm using simulations that such a permutation-based assessment produces the expected type-I error rate (Supplemental Fig. S13E).

**Figure 5.**
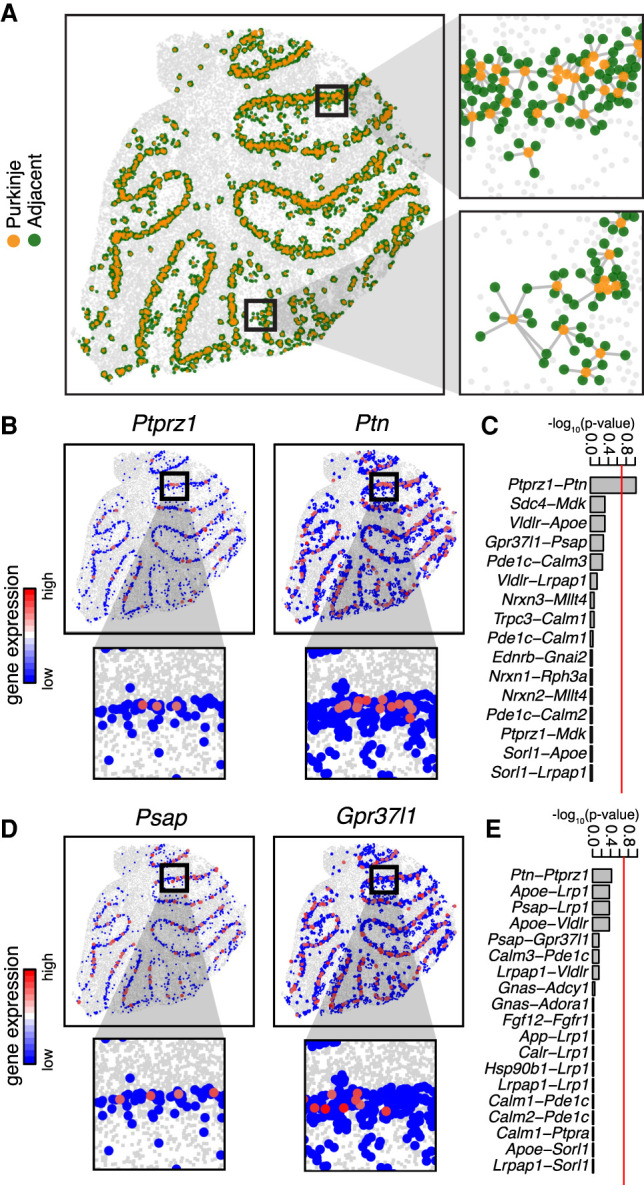
MERINGUE identifies putative cell–cell communication in the cerebellum using Slide-seq data. (*A*) Adjacency relationship between Slide-seq beads. Orange dots correspond to beads previously annotated as corresponding to the Purkinje layer. Green dots correspond to beads that are spatially adjacent. Gray lines connect each bead with its spatial neighbors and is agnostic to bead density. (*B*, *left*) Expression of receptor *Ptprz1* in beads annotated to correspond to the Purkinje layer. (*Right*) Expression of corresponding ligand *Ptn* in spatially adjacent beads. Same select region highlighted. (*C*) Bar plot of −log_10_(adjusted *P*-value) for the inter-cell-type spatial cross-correlation statistic of all receptors in Purkinje layer beads versus ligands in the spatially adjacent beads. Red line indicates alpha = 0.2 multiple testing corrected significance threshold. (*D*, *left*) Expression of ligand *Psap* in beads annotated to correspond to the Purkinje layer. (*Right*) Expression of corresponding receptor *Gpr37l1* in spatially adjacent beads. Same select region highlighted as *D*. (*E*) Bar plot of −log_10_(adjusted *P*-value) for the inter-cell-type spatial cross-correlation statistic of all ligands in Purkinje layer beads versus receptors in the spatially adjacent beads.

We first applied our approach to identify gene expression patterns that may be indicative of putative cell–cell communication between cells on beads corresponding to the Purkinje layer with cells on spatially adjacent beads in Slide-seq data of the mouse cerebellum (Fig. 5A). We used a set of more than 2500 known receptor-ligand pairs previously supported by orthogonal biological validations (Ramilowski et al. 2015). Restricting to well-detected (CPM > 0 in more than 30 cells) receptor genes in the Purkinje layer beads and well-detected ligand genes in the spatially adjacent beads, we applied MERINGUE to test for significant spatial cross-correlation between all receptor and ligand gene pairs. We identified statistically significant inter-cell-type spatial cross-correlation between expression of protein tyrosine phosphatase, receptor type Z, polypeptide 1 (*Ptprz1* [also known as *PTPζ*]) in beads corresponding to Purkinje layer and expression of its ligand *Ptn* (secreted growth factor pleiotrophin) in spatially adjacent beads (Fig. 5B,C). *Ptprz1* has been previously identified to be expressed by Purkinje neurons, whereas *Ptn* has been previously identified to distribute along Bergmann glial fibers in postnatally developing cerebellum (Matsumoto et al. 1994). Although this Slide-seq data set does not provide single-cell resolution, we confirm significant coexpression of *Ptprz1* with Purkinje cell specific promoter *Pcp2* (Fisher's exact test *P*-value = 2.3 × 10^−18^), suggestive that the *Ptprz1* expression may be attributed to Purkinje cells within the Purkinje layer beads. Likewise, we confirm significant coexpression of *Ptn* with *Slc1a3* (glutamate aspartate transporter [also known as GLAST]), a glutamate transporter expressed by Bergmann glia (Fisher's exact test *P*-value = 4.5 × 10^−36^). In contrast, restricting to well-detected ligand genes in the Purkinje layer beads and well-detected receptor genes in the spatially adjacent beads (Fig. 5D,E), we do not identify significant spatial cross-correlation between any receptor and ligand gene pairs, including between *Ptn* expression in beads corresponding to Purkinje layer and *Ptprz1* expression in spatially adjacent beads, indicative of the cell type specificity of inferred receptor-ligand interactions. Previous studies with cerebellar slice culture systems have shown that PTN-PTPRZ1 signaling is involved in the morphogenesis of Purkinje dendrites (Tanaka et al. 2003). The identification of such putative cell–cell communication between Bergmann glia and Purkinje cells may be suggestive of the potential for glial signals to actively regulate neuronal function and contribute to sustained plasticity in adult brains (Barres 2008).

**Figure 6.**
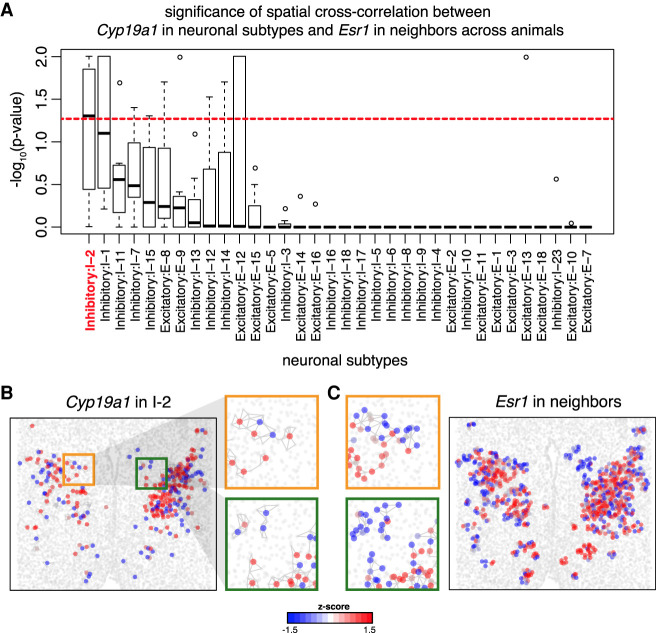
MERINGUE systematically and quantitatively evaluates for putative cell–cell communication for neuronal subtypes in the preoptic region using MERFISH data. (*A*) Distribution of −log_10_(*P*-values) for the spatial cross-correlation between *Cyp19a1* expression in neuronal subtypes and *Esr1* expression in adjacent cells across animals. Boxes in the box plot denote the median values and inner quartile ranges (IQR) and whiskers denote 1.5 × IQR with additional outliers represented as points. Red dotted line is the alpha = 0.05 significance threshold. Generally, inhibitory neuron subtype I-2 shows significant spatial cross-correlation between *Cyp19a1* expression and *Esr1* expression in adjacent cells in a manner that is consistent across animals. (*B*) *Cyp19a1* expression in I-2 neurons in one tissue slice in one animal with red indicating high expression and blue indicating low expression. Representative slice and animal shown. Select areas are highlighted in the zoom-in. (*C*) *Esr1* expression in cells neighboring I-2 neurons in one tissue layer in one animal, with red indicating high expression and blue indicating low expression. Representative slice and animal shown. The same select areas as *B* are highlighted in the zoom-in.

We next sought to identify gene expression patterns that may be indicative of putative cell–cell communication between cell types using single-cell resolution MERFISH data of the preoptic region. Previously, Moffitt et al. (2018) visually noted that *Cyp19a1* (also known as aromatase) enriched inhibitory I-2 neurons displayed substantial spatial overlap with estrogen receptor (*Esr1*) enriched neuronal subtypes. CYP19A1 is an enzyme that converts testosterone to estrogen, thereby modulating steroid hormone signaling in the preoptic region. The spatial organization of these CYP19A1-enriched neuronal subtypes with ESR1-enriched cells suggest that estrogen synthesized by these CYP19A1-expressing neurons may be interacting with estrogen receptors on spatially adjacent ESR1-expressing cells in a paracrine manner. To quantitatively assess to putative cell–cell communication between neuronal subtypes and spatially neighboring cells via such paracrine signaling, we applied MERINGUE to assess for significant spatial cross-correlation between *Cyp19a1* expression in all neuronal subtypes and *Esr1* expression in spatially adjacent neurons. Indeed, we are able to identify statistically significant spatial cross-correlation between *Cyp19a1* expression in I-2 neurons and *Esr1* expression in spatially adjacent neurons in a manner that is consistent across tissue layers and reproducible across animals (Fig. 6A). In contrast, neuronal subpopulations I-13 also express *Cyp19a1* but do not show significant spatial cross-correlation with *Esr1* in surrounding cells (Fig. 6B,C; Supplemental Fig. S14A). Furthermore, we also apply MERINGUE to test for spatial cross-correlation between *Cyp19a1* expression in all neuronal subtypes and androgen receptor (*Ar*) expression in spatially adjacent neurons and do not identify consistently significant associations (Supplemental Fig. S14B), thereby highlighting the nonrandomness of the *Esr1* juxtaposition. This thus highlights MERINGUE's potential to quantitatively and systematically identify complementary gene expression patterns that may be indicative of cell–cell communication.

## Discussion

Spatially resolved transcriptomic measurements demand computational approaches to identify and characterize significant spatial gene expression heterogeneity. Here, we presented MERINGUE as a density-agnostic approach to characterize spatially heterogeneous gene expression by identifying genes with spatially autocorrelated expression and gene pairs with spatially cross-correlated expression. We validated our approach by analyzing spatially resolved transcriptomic data from both sequencing and imaging-based methods in 2D and 3D to recover known biologically relevant spatial patterns. Our analysis of the mouse preoptic region by MERFISH revealed sexually dimorphic spatial organization of *Tacr1* expression in excitatory E-8 neurons and identified additional neuronal subpopulations within inhibitory I-2 and I-11 neurons with spatially distinct organization that may play roles in murine sexual behavior. MERINGUE is highly scalable and computationally efficient compared to previous spatial analysis methods (Supplemental Fig. S15). Furthermore, MERINGUE is robust to spatial variations in cellular density and can thus better accommodate nonuniform cellular densities common in tissues.

In comparison with previously published spatial gene expression analysis methods, although MERINGUE identifies and groups spatially heterogeneous genes into primary spatial patterns, it does not interpret identified spatial patterns based on predefined aggregated or alternating spatial patterns. In this manner, we find MERINGUE to be complementary to previously published spatial gene expression analysis methods in characterizing the spatial patterns of spatially heterogeneous genes. Likewise, we find MERINGUE to be complementary to expression-based clustering analysis to identify additional aspects of spatial heterogeneity within cell clusters or shared spatial gradients across cell clusters. In addition, in analyzing spatially resolved single-cell gene expression data sets obtained from different technologies, MERINGUE may also be applied in combination with different normalization and error model schemes such as cell volume-based normalization for imaging data (Moffitt et al. 2018) and cell density normalization for ST data (Saiselet et al. 2020). Furthermore, for zero-inflated transcriptomics measurements, additional dropout error modeling or imputation of dropouts may be applied before MERINGUE analysis (Kharchenko et al. 2014; Hou et al. 2020).

Finally, although MERINGUE uses spatial cross-correlation analysis to identify gene expression patterns that may be indicative of putative cell–cell communication, such inference is based on spatial proximity, which restricts inferred interactions to short-range interactions or chemical cues. This is limiting for tissues such as the mammalian brain, where neuronal communication and interactions often span long distances because of long axons and dendritic processes. We anticipate that additionally combining single-cell transcriptomics profiling with neuronal tracing could derive new binary weight matrices that would fit into MERINGUE's analysis framework, enabling study of more comprehensive cell–cell interactions in a spatially resolved manner. Likewise, in the future, computational approaches such as MERINGUE, in combination with systematic biological perturbations, can help elucidate the mechanisms responsible for these spatial patterns and enhance our understanding of the spatial organization of and communications between cell types and cell states within tissues.

## Methods

### MERINGUE approach

#### Data preprocessing and quality control

Data must be corrected for sequencing depth differences and other technically driven variation of expression magnitude before MERINGUE. Counts per million (CPM) normalization without log transforming was applied to all spatially resolved transcriptomic data sets. For MERFISH data, RNA counts were normalized per cell by the imaged volume of each cell per the originally published analysis (Moffitt et al. 2018).

#### Adjacency weight matrix

Given a set of spatial positional coordinates for spatially resolved data sets, such as cells, MERINGUE represents these cells as connected neighborhoods in space using an adjacency weight matrix *W*, where

$$W_{ij}=\{\begin{array}{cc} 1\;\; & {\mathrm{if}\;\mathrm{cell}}_{i}\;{\mathrm{and}\;\mathrm{cell}}_{j}\;\mathrm{are}\;\mathrm{adjacent}\;\\ 0\; & {\mathrm{if}\;\mathrm{cell}}_{i}{\;\mathrm{and}\;\mathrm{cell}}_{j}\;\mathrm{are}\;\mathrm{not}\;\mathrm{adjacent}.\; \end{array}$$

Cells are defined as adjacent using Delaunay triangulation. The Delaunay triangulation of a discrete set of points (in this case, cells in space) is equivalent to the Voronoi diagram for the same set of points (Okabe et al. 1992). This approach is thus equivalent to defining cells as adjacent if they have Voronoi polygons, as inferred from Voronoi tessellation, that share an edge. For biological interpretability, adjacency relationships beyond a certain spatial distance can also be ignored. Delaunay triangulation can also accommodate 3D data.

#### Identifying significantly spatially heterogeneous genes

We define spatially heterogeneous genes as genes with uneven, often aggregated or patterned, spatial distribution of expression magnitudes. MERINGUE identifies such spatially heterogeneous genes by computing Moran's *I* (Moran 1950)

$$I=\frac{N}{\sum_{i}^{N}\sum_{j}^{N}W_{ij}}\frac{\sum_{i}^{N}\sum_{j}^{N}W_{ij}(x_{i}-\bar{x})(x_{j}-\bar{x})}{\sum_{i}^{N}{\left(x_{i}-\bar{x}\right)}^{2}}$$

for each gene given its normalized gene expression vector *x* across a population of *N* cells using the adjacency weight matrix *W* described previously to detect for positive spatial autocorrelation.

The expected value of *I* under the null hypothesis of no spatial autocorrelation can be solved by computing the first moment (Getis 1995) and simplified to

$$E(I)=\frac{-1}{N-1}.$$

Likewise, variance can be derived using the second moment and simplified to

$$\text{Var}(I)=\frac{N^{*}S_{4}+{S_{3}}^{*}S_{5}}{(N-1)(N-2)(N-3)W^{2}}-{\left(\frac{-1}{N-1}\right)}^{2},$$

where

$$S_{1}=\frac{1}{2}\overset{N}{\underset{i}{\sum }}\overset{N}{\underset{j}{\sum }}{\left(W_{ij}+W_{\;ji}\right)}^{2}$$

$$S_{2}=\overset{N}{\underset{i}{\sum }}{\left(\overset{N}{\underset{j}{\sum }}W_{ij}+\overset{N}{\underset{j}{\sum }}W_{\;ji}\right)}^{2}$$

$$S_{3}=\frac{\frac{\sum_{i}^{N}{\left(x_{i}-\bar{x}\right)}^{4}}{N}}{{\left(\frac{\sum_{i}^{N}{\left(x_{i}-\bar{x}\right)}^{2}}{N}\right)}^{2}}$$

$$S_{4}={\left(N^{2}-3^{*}N+3\right)}^{*}S_{1}-N^{*}S_{2}+3^{*}W^{2}$$

$$S_{5}={\left(N^{2}-N\right)}^{*}S_{1}-2^{*}N^{*}S_{2}+6W^{2}$$

$$W=\overset{N}{\underset{i}{\sum }}\overset{N}{\underset{j}{\sum }}w_{ij}.$$

We implemented these calculations in C++ using Rcpp (Eddelbuettel and François 2011).

In a given data set, we evaluated all genes for spatial heterogeneity and applied the Benjamini–Hochberg procedure to correct for multiple testing and control for false discovery (Benjamini and Hochberg 1995).

We assumed here that the expression magnitudes represented by each neighborhood is comparable such that observed differences in gene expression levels across neighborhoods are not the result of different sequencing depths or other technical confounders. In this manner, *x* must already be normalized to control for variability in sequencing depth or other technical confounders, where appropriate, before analysis with Moran's *I*. Likewise, because Moran's *I* is not defined for constant signals, genes without any expression variability were omitted from analysis.

If the data are produced by a mechanism that inherently induces some autocorrelation, such as high variability between spatially segregated batches or presence of noisy hotspots, then such a null hypothesis would not be appropriate, and evaluation of significance must be performed using permutation. We showed at least for a random subset of genes in our tested data sets that the null hypothesis is appropriate and thus results in essentially identical *P*-values regardless of approach (Supplemental Fig. 2B).

#### Characterizing the scale of significantly spatially heterogeneous genes

For a given gene *x* identified as significantly spatially heterogeneous, MERINGUE next quantifies the scale of the spatial pattern by calculating the local indicators of spatial association (Anselin 1995) (LISA) for each neighborhood: *i*

$$I_{i}=N\frac{\left(x_{i}-\bar{x})\sum_{j}^{N}W_{ij}(x_{j}-\bar{x}\right)}{\sum_{i}^{N}{\left(x_{i}-\bar{x}\right)}^{2}}.$$

LISA relates to Moran's *I* via

$$I=\overset{N}{\underset{i}{\sum }}\frac{I_{i}}{N},$$

and as such, LISA also contains a closed form that can be solved for its expected value and standard deviation under the null hypothesis of no spatial autocorrelation. We defined the scale of a gene's spatial pattern as the percentage of cells with a LISA that is statistically significant, that is, has a *P*-value below an alpha threshold (default: 0.05). Downstream analyses can be restricted to spatially heterogeneous genes of a sufficient scale, defined by default as 5% of cells.

Again, these calculations were implemented in C++ using Rcpp (Eddelbuettel and François 2011).

For visualization purposes, we further implemented a signed LISA score:

$$sI_{i}=sign{\left(x_{i}-\bar{x}\right)}^{*}\;N\frac{\left(x_{i}-\bar{x})\sum_{j}^{N}w_{ij}(x_{j}-\bar{x}\right)}{\sum_{i}^{N}{\left(x_{i}-\bar{x}\right)}^{2}}.$$

#### Primary pattern determination using spatial cross-correlation analysis

After identifying significantly spatially heterogeneous genes of a sufficient scale, MERINGUE groups these genes into primary spatial patterns. We calculate a spatial cross-correlation index (SCI) between all pairs of these genes. For *N* cells, gene *x*, and gene *y*, the SCI can be calculated as

$$\mathrm{SCI}=\frac{N}{2\sum_{i}^{N}\sum_{j}^{N}W_{ij}}\frac{\sum_{i}^{N}\sum_{j}^{N}W_{ij}(x_{i}-\bar{x})(y_{j}-\bar{y})}{\sqrt{\sum_{i}^{N}{\left(x_{i}-\bar{x}\right)}^{2}}\sqrt{\sum_{j}^{N}{\left(y_{j}-\bar{y}\right)}^{2}}}.$$

The SCI for all pairs of genes forms a spatial cross-correlation matrix, which we used as the basis for hierarchical clustering. Clusters of genes were then identified using dynamic tree cutting (Langfelder et al. 2008) such that highly spatially cross-correlated genes fall into the same clusters, thus comprising the primary spatial patterns. By default, the hybrid dynamic tree cutting approach was used.

We visualized these primary patterns by interpolating across spatial regions not covered by cells using Akima interpolation (Akima 1996a,b).

#### Spatially informed clustering

To identify spatially distinct but transcriptionally similar subpopulations, we began with graph-based expression clustering. Specifically, we constructed a *k*-nearest neighbor graph on the reduced principal components space derived from normalized gene expression. In such a graph, each node is a cell and they are connected with an edge if they are among the *k*-nearest neighbors based on transcriptional similarity. To introduce spatial information, we weighted the edges of the graph based on the geodesic spatial distance between the two nodes’ cells. The geodesic spatial distance is computed based on the adjacency matrix *W* where two cells would have a spatial distance of 1 if they are neighbors or 2 if they are neighbors of neighbors and so forth. We then transformed the spatial distance into a weight that is inversely proportional to the distance such that cells closer together (i.e., with a small distance) will be given higher weight and cells farther apart (i.e., large distance) will be given a smaller weight: weight = (1/distance + *α*) + *β*, where *α* and *β* are pseudocounts to guard against excessively large and small weights, respectively. By default, we used *α* = *β* = 1, though the unit and magnitude of both *α* and *β* will depend on the unit and magnitude of distance. We then apply Louvain graph-based clustering to the resulting weighted graph (Phyu and Myat Min 2019).

#### Inference of cell–cell communication using inter*-*cell*-*type spatial cross*-*correlation analysis

To infer cell–cell communication between spatially colocalized cell types, MERINGUE focuses on identifying complementary gene expression patterns between known receptor-ligand pairs (Ramilowski et al. 2015).

For each receptor-ligand pair, we computed an inter-cell-type spatial cross-correlation (*i*SCI) between expression of receptor *x* for the *N* cells of cell type A and the expression of ligand *y* for the *M* cells of cell type B:

$$i\mathrm{SCI}=\frac{N+M}{2\sum_{i}^{A}\sum_{j}^{B}W_{ictij}}\frac{\sum_{i}^{N}\sum_{j}^{M}W_{ictij}(x_{i}-\bar{x})(y_{j}-\bar{y})}{\sqrt{\sum_{i}^{A}{\left(x_{i}-\bar{x}\right)}^{2}}\sqrt{\sum_{j}^{B}{\left(y_{j}-\bar{y}\right)}^{2}}}.$$

Here, the inter-cell type adjacency weight matrix *W*_*ict*__*ij*_ = 1 if a cell of cell type A and a cell of cell type B are inferred to be adjacent or vice versa, and *W*_*ict*__*ij*_ = 0 otherwise, to capture only spatial cross-correlation patterns between the two cell types.

We assessed statistical significance by comparing the observed *i*SCI with the likelihood of observing such an extremely positive value under a permutation-based random labeling model randomly permuting cell labels. To enhance computational efficiency, we allowed for parallelization across multiple cores and used an adaptive permutation testing approach whereby receptor-ligand pairs are first assessed for significance with 100 permutations by default, and putatively significant hits with permutation *P*-values < 1/100 are then reassessed with 1000 permutations and so forth. Additional gene pairs with known interacting products such as hormone-receptors can also be evaluated by this approach.

#### Interactive application

An interactive application built on Shiny (Chang et al. 2020) can be launched directly from R sessions to enable interactive visual exploration of MERINGUE results and statistics.

## Supplementary Material

Supplemental Material

## Software availability

MERINGUE is programmed in C++ and available as an open-source R software package (R Core Team 2020) with the source code available in the Supplemental Material and on GitHub (https://github.com/JEFworks-Lab/MERINGUE). Additional documentation and tutorials are available (https://JEF.works/MERINGUE).
