# Supplementary material for: Characterizing spatial gene expression heterogeneity in spatially resolved single-cell transcriptomic data with nonuniform cellular densities
Source: Genome Res. 2021 Oct;31(10):1843–55. doi: 10.1101/gr.271288.120 (PMC8494224; doi:10.1101/gr.271288.120)
Supplement: Supplemental Material [file supp_31_10_1843__DC1.html]

Characterizing spatial gene expression heterogeneity in spatially resolved single-cell transcriptomic data with nonuniform cellular densities — Supplemental Material 

# Characterizing spatial gene expression heterogeneity in spatially resolved single-cell transcriptomic data with nonuniform cellular densities

## Supplemental Material

- SupplementaryMaterials-clean-vF.pdf
- Supplemental\_Table\_S1.xlsx
- Supplemental\_Table\_S2.xlsx
- Supplemental\_Table\_S3.xlsx
- Supplemental\_Table\_S4.xlsx
- Supplemental\_Table\_S5.xlsx
- Supplemental\_Software\_MERINGUE\_1.0.tar.gz
