# Supplementary material for: Characterizing spatial gene expression heterogeneity in spatially resolved single-cell transcriptomic data with nonuniform cellular densities
Source: Genome Res. 2021 Oct;31(10):1843–55. doi: 10.1101/gr.271288.120 (PMC8494224; doi:10.1101/gr.271288.120)
Supplement: Supplemental Material [file supp_gr.271288.120_SupplementaryMaterials-clean-vF.pdf]

Supplemental Material for "Characterizing spatial gene expression heterogeneity in spatially resolved single-cell transcriptomics data with non-uniform cellular densities"

A. Supplemental Methods

B. Supplemental Figures Legends

Supplemental Figure S1. Challenges imposed by non-homogenous cell-densities in spatial analysis.
Supplemental Figure S2. Parameter and design justifications.
Supplemental Figure S3. Additional analysis of Spatial Transcriptomics (ST) data of the mouse olfactory bulb (MOB).
Supplemental Figure S4. Analysis of SlideSeq of the mouse cerebellum.
Supplemental Figure S5. Comparison of MERINGUE to previously published methods for analyzing spatially resolved transcriptomics data using ST data of the MOB.
Supplemental Figure S6. Comparison of MERINGUE to previously published methods in analyzing spatial transcriptomics data with uniform and non-uniform spot densities.
Supplemental Figure S7. Additional analysis of aligned ISH data of the *Drosophila melanogaster* embryo.
Supplemental Figure S9. Additional examples of aspects of spatial heterogeneity in the preoptic region.
Supplemental Figure S10. Consistency of spatial patterns in the preoptic region across female and male animals.
Supplemental Figure S11. Simulation of spatially informed clustering.
Supplemental Figure S12. Transcriptional characterization of spatially informed clusters in the preoptic region.
Supplemental Figure S13. Simulation of inter-cell-type spatial cross correlation.
Supplemental Figure S14. Additional examples of putative cell-cell communication in the preoptic region.
Supplemental Figure S15. Comparison of Moran's I as implemented in spdep versus MERINGUE in evaluating 2000 genes across 260 spots in the MOB dataset

C. Supplemental References

A. Supplemental Methods

MERINGUE analysis of Spatial Transcriptomics of the mouse olfactory bulb (MOB)

Data was downloaded from https://www.spatialresearch.org/ for the MOB Replicate 11 sample that was described in the original publication (Ståhl et al. 2016).

Beginning with 262 probe spots and 15928 genes, we filtered out spots with fewer than 100 genes detected and filtered out genes with fewer than 100 reads across all spots resulting in 260 probe spots and 7365 genes. Counts were normalized to counts per million (CPM) values for downstream analysis.

For the expression-based analysis, we performed principal components analysis (PCA) on the performed on the $\log_{10}$ transform CPM values with pseudo count of 1. To identify transcriptional clusters, we performed Louvain clustering on a *k*-nearest neighbor graph constructed from the top 5 PCs with k=30. We manually annotated identified clusters as different cell layers to best correspond to the original publication (Ståhl et al. 2016). We visualized the results using t-stochastic neighbor embedding (tSNE) on the top 5 PCs.

For differential expression analysis, we use ANOVA testing to identify genes with significant expression variability across the annotated cell layers (adjusted p-value < 0.05).

For the spatial analysis, we filtered out adjacency relationships beyond a Euclidean distance of 2 (corresponding approximately 300 microns) to minimize long-range adjacency relationships towards the edge of the sample. We restricted to significantly (adjusted p-value < 0.05) spatially heterogeneous genes driven by more than 5% of spots. We grouped these significantly spatially variable genes into primary spatial patterns using hierarchical clustering with the 'ward.D' approach and deepSplit=4 parameter for dynamic tree cutting using the hybrid approach (Langfelder et al. 2008). For visualization, we interpolated expression patterns with a binSize of 50.

For the analysis of olfactory receptor (*Olfr*) genes, we identified summing the counts of all *Olfr* genes detected in each spot and assessed the summed expression for spatial heterogeneity.

MERINGUE analysis of aligned *In Situ* hybridization (ISH) of the *Drosophila melanogaster* embryo

Data was downloaded from DVEX (Karaiskos et al. 2017) at https://shiny.mdc-berlin.de/DVEX/ for the continuous in situ expression matrix from the BDTNP (Fowlkes et al. 2008).

We analyzed the *in situ* expression of 84 genes across the 3039 aligned stage 6 *Drosophila melanogaster* embryonic cell locations. For the spatial analysis, we used the 3D x, y, z coordinates and filtered out adjacency relationships beyond a 3D Euclidean distance of 10 to minimize long-range adjacency relationships towards the edge of the sample. We restricted to significantly (adjusted p-value < 0.05) spatially variable genes driven by more than 5% of locations. We grouped these significantly spatially variable genes into primary spatial patterns using hierarchical clustering with the 'ward.D' approach and deepSplit=2 parameter for dynamic tree cutting. For visualization, we interpolated expression to the whole embryo with a binSize of 50.

For expression-based clustering, to recapitulate results from (Karaiskos et al. 2017), we performed Louvain clustering on a *k*-nearest neighbor graph constructed from the top 20 PCs with k=150. For spatially aware clustering, we apply Louvain clustering to this same *k*-nearest neighbor graph but with edge weights using the same 3D adjacency weight matrix as the spatial analysis with $\alpha = \beta = 0.01$.

MERINGUE analysis of multi-section Spatial Transcriptomics of breast cancer tissue

Data was downloaded from https://www.spatialresearch.org/ for the 4 breast cancer sections that was described in the original publication (Ståhl et al. 2016).

Beginning with 4 breast cancer tissue sections, we concatenated expression matrices based on genes detected in all tissue layers, resulting in 13360 genes across 1031 probe spots. We further filtered out spots with fewer than 100 genes detected and filtered out genes with fewer than 100 reads across all

spots resulting in 6214 genes across 1029 probe spots. Counts were normalized to counts per million (CPM) values for downstream analysis.

For each individual breast cancer tissue layer, we first performed spatial analysis by constructing an adjacency weight matrix based on probe spots within the individual layer and filtered out adjacency relationships beyond a Euclidean distance of 2. We restricted to significantly (adjusted p-value < 0.05) spatially variable genes driven by more than 5% of spots in that tissue layer.

To identify patterns consistent across tissue layers, we manually rotated and centered spatial spot coordinates to enable the construction of a cross-layer adjacency weight matrix based on the k mutual nearest neighbors of probe spots across aligned layers with K = 3. We then identified significantly (adjusted p-value < 0.05) spatially variable genes driven by more than 1.25% of all spots.

MERINGUE analysis of SlideSeq data of the mouse cerebellum

Data was downloaded for Puck_180819_12 that was described in the original publication (Rodriques et al. 2019).

We focus our analysis on beads within the Purkinje layer of the mouse cerebellum, which was annotated as AnalogizerClusterAssignmentsOriginal=6 in the original publication, resulting in 1589 spots. We further filtered out beads with fewer than 100 genes detected and filtered out genes with fewer than 100 reads across all beads resulting in 191 genes across 883 beads. Counts were normalized to counts per million (CPM) values for downstream analysis.

For the spatial analysis, we filtered out adjacency relationships beyond a Euclidean distance of 250. We restricted to significantly (adjusted p-value < 0.05) spatially variable genes driven by more than 5% of beads.

For the inference of cell-cell communication, we focus on known receptor-ligand pairs (Ramilowski et al. 2015). From the adjacency matrix, we identify beads spatially adjacent to the Purkinje layer beads. We restrict to testing receptor and ligand pairs that are detected (> 0 expression) in at least 30 beads in the Purkinje and neighbor beads respectively and vice versa. Observed iSCI were evaluated for statistical significance using adaptive permutation testing for 100, 1000, and 10000 permutations. We applied the Benjamini-Hochberg procedure for multiple testing correction to account for all receptor and ligand pairs tested.

MERINGUE analysis of MERFISH data of the mouse hypothalamic preoptic region

Data was downloaded from the original publication (Moffitt et al. 2018).

For the spatial analysis, we focused on each cell-type and subtype as annotated in the original publication. Expression levels were normalized per cell by the imaged volume of each cell per the originally published analysis (Moffitt et al. 2018). For each individual tissue layer in each animal, we first performed spatial analysis on cell-types and subtypes that are present with more than 30 cells, constructed an adjacency weight matrix, and filtered out adjacency relationships beyond a Euclidean distance of 250 to minimize long-range adjacency relationships towards the edge of the sample. Due to concerns of subtype annotation errors potentially driving spatial patterns, we require significantly spatial variable genes to be driven by more than 10% of cells. In this manner, if certain subtypes contain a few mis-annotated cells that are truly from another transcriptionally and spatially distinct subtype, we would not mis-interpret the mis-annotation as spatial heterogeneity. Further, due to concerns of spatially patterned misidentification, we further require cells driving identified spatially heterogeneous genes to express the genes at a minimum expression magnitude calculated from the 95th percentile of blank control misidentification rate. In this manner, low expression from spatially patterned misidentification would not be mis-interpreted as spatial heterogeneity. In addition, we required patterns to be significantly consistent across tissue sections. To identify patterns consistent across tissue sections, for each animal, we ordered tissue sections by the annotated Bregma position per the original publication. We centered the x and y-positions to approximately align tissue sections. We then constructed a cross-layer adjacency weight matrix based on the k mutual nearest neighbors of cells across aligned layers with k = 3. Finally, we

considered a gene as spatially heterogeneous if it was identified to exhibit significant spatial heterogeneity both within individual tissue sections and across tissue sections in at least 25% of evaluated animals.

To identify potential sexually dimorphic differences in gene expression patterns, visually evaluated identified spatially heterogeneous genes to identify *Nos1* expressing in Inhibitory neuron I-11 as a potential candidate. To test for statistical differences, for each animal, we quantified the fraction of *Nos1*+ I-11 neurons by comparing I-11 neurons expressing *Nos1* versus all I-11 neurons. We defined I-11 neurons expressing *Nos1* as those with *Nos1* detected at above the 95-percentile blank expression rate = 0.1. We tested for differences between the fraction of *Nos1*+ I-11 neurons between female and male naïve mice for a representative tissue section with the most abundant I-11 neurons using a Student's t-test. Likewise, we systematically evaluated genes for spatial heterogeneity separately for naïve male and female mice using the same standards above and identified *Tacr1* expression in Excitatory neuron E-8 as being significantly spatially heterogeneous in male but not female naïve animals. We used LISA to quantify the scale of spatial heterogeneity for *Tacr1*. We tested for differences between the scale of *Tacr1* spatial heterogeneity in E-8 neurons between female and male naïve mice for a representative tissue section with the most abundant E-8 neurons using a Student's t-test.

For expression-based clustering, we focus on only inhibitory neurons in animal FN7, tissue section brain_pos=9. We restricted analysis to neuronal subtypes that are present with more than 100 cells. To recapitulate results from (Moffitt et al. 2018), we performed Infomap clustering on a k-nearest neighbor graph constructed from the top 50 PCs with k=50. For spatially informed clustering, due to bilateral symmetry in the preoptic region, we constructed the adjacency matrix W on transformed x-positions where $x' = abs(x - \overline{x})$ in order to accommodate the known symmetry about the y-axis. We again applied Infomap clustering to this same k-nearest neighbor graph but with edge weights using the adjacency matrix W on transformed x-positions with $\alpha = \beta = 1$. Our spatially informed clustering identified additional clusters splitting Inhibitory neurons subtype I-11 and I-2. To generalize the spatially aware clustering to all animals, we trained a linear discriminant classifier to map from gene expression to the identified spatially aware clustering annotations for I-11 and I-2 separately and applied the classifier to I-11 and I-2 in all animals separately. This analysis was repeated for excitatory neurons. To test for differential activation between I-2 subtypes identified by spatially informed clustering, we evaluated the fraction of activated neurons for each I-2 subtype. We define activated neurons as those with *cFo*s expression > 0.1.

For the inference of cell-cell communication, we specifically focus testing for inter-cell-type spatial cross-correlation between *Cyp19a1* with *Esr1* or *Ar*. For each tissue layers in each animal, we restricted analysis to neuronal subtypes that are present with more than 100 cells. We construct an adjacency weight matrix based on all annotated cells within the tissue layer, excluding cells annotated as ambiguous from the original publication.

Comparing MERINGUE to previously published spatial analysis methods

We compare MERINGUE to previously published spatial analysis methods SpatialDE v1.1.3, Trendsceek v1.0.0, and SPARK v1.1.0. Trendsceek and SPARK were run from within R while SpatialDE was run using R's reticulate package to interface with Python.

To compare each method's results on the MOB dataset, we restricted to the same filtered set of 260 cells and 7365 genes. For MERINGUE and SpatialDE, CPM normalized expression values were used. For SPARK, counts were used as SPARK performs internal library size normalization. Trendsceek could not complete in a reasonable amount of time and was omitted from comparison. To evaluate the overlap between identified significantly spatially heterogenenous genes, MERINGUE results were filtered for genes with adjusted p-value < 0.05 driven by > 5% of spots, SpatialDE results were filtered for genes with a adjusted p-value threshold < 0.05, and SPARK results were filtered with an adjusted p-value < 0.05 combined across all default evaluated kernels.

To compare the runtime and memory usage of each method in identifying significantly heterogeneous genes, we used the 10X Visium dataset of the mouse coronal brain section downloaded

from https://www.10xgenomics.com/products/spatial-gene-expression. To evaluate runtime as a function of spots, we downsampled the data to a constant set of 1000 genes and varied the number of spots from 125, 250, 500, to 1000 and evaluated the runtime of each method. Likewise, to evaluate runtime as a function of genes, we downsampled the data to a constant set of 1000 spots and varied the number of genes from 125, 250, 500, to 1000 and evaluated the runtime of each method. We evaluated memory usage as the amount of memory allocated and then subsequently released after the running lines corresponding to each method, excluding the amount of memory necessary to create or store the positional and gene expression matrices. For Trendsceek, we limited to 100 permutations and only evaluated performance for the two smallest datasets due to runtime constraints. For SPARK and Trendsceek, we limited computation to a single core for comparison purposes. SpatialDE was omitted from comparison due to concerns that R-to-Python conversion differences introduced non-comparable runtime and memory usage.

Comparing the implementation of Moran's I in MERINGUE with spdep

spdep (Bivand et al, 2013) also has an implementation of Moran's I in R. We compared the implementation of Moran's I in the spdep library (v1.1-7) to the implementation in MERINGUE in evaluating 2000 genes across 260 spots in the MOB dataset. We provide identical adjacency matrices to confirm that both implementations yielded equivalent I statistics (Spearman correlation R=1). We also evaluated 2000 genes across 260 spots in the MOB dataset to compare runtime per gene. Moran's I in MERINGUE is written in C++ whereas the spdep implementation is written in native R.

Evaluating robustness to changes in cellular density

To evaluate robustness to changes in cellular density, we used the MOB dataset's spatial coordinates, centering and raising 1.1 to the power of the x-coordinates in order to induce non-uniform spot densities. We then compared each method's resulting $-\log_{10}$(adjusted p-values) or $-\log_{10}$(combined adjusted p-values) where appropriate under the uniform and non-uniform density cases.

Potential limitations of MERINGUE

MERINGUE builds on Moran's I, which others have noted may result in inflated P-values, which could lead to false positives (Sun et al. 2020). However, we find MERINGUE's additional filtering criterions using LISA to restrict to spatial patterns driven by a certain proportion of cells can minimize false positives (Supplemental Fig. 2C). Furthermore, conservative P-value corrections scores can help minimize such false positives.

MERINGUE requires that gene expression magnitudes within an individual spatially resolved transcriptomics dataset have been either collected in a single batch or have been corrected for batch effects. Therefore, expression-level batch correction may be applied prior to analysis with MERINGUE to ensure that identified spatial patterns are not driven by batch (Johnson et al. 2007).

B. Supplementary Figures

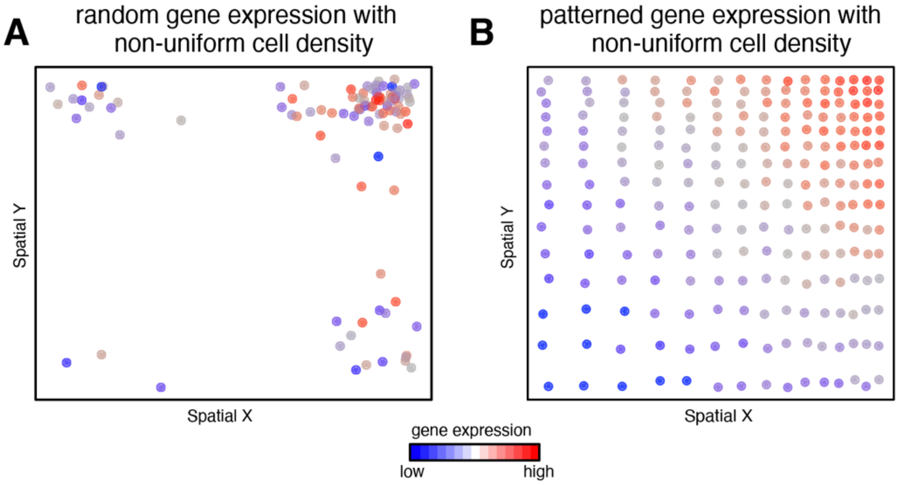

Supplemental Figure S1. Challenges imposed by non-homogenous cell-densities in spatial analysis. We simulate cells in space for illustrative purposes. Each point is a cell. Red indicates high expression, while blue indicates low expression of a simulated gene. **A.** An example of random gene expression with non-uniform cell density. Cells are denser in the top right corner. Gaussian randomly distributed gene expression is simulated across cells. Although gene expression is randomly distributed across all cells, higher gene expression is observed by chance in the denser region. Methods that fail to take into consideration differences in cell density may mistake such density-confounded random gene expression as spatial heterogeneity due to density aggregation. **B.** An example of patterned gene expression confounded by cell density.

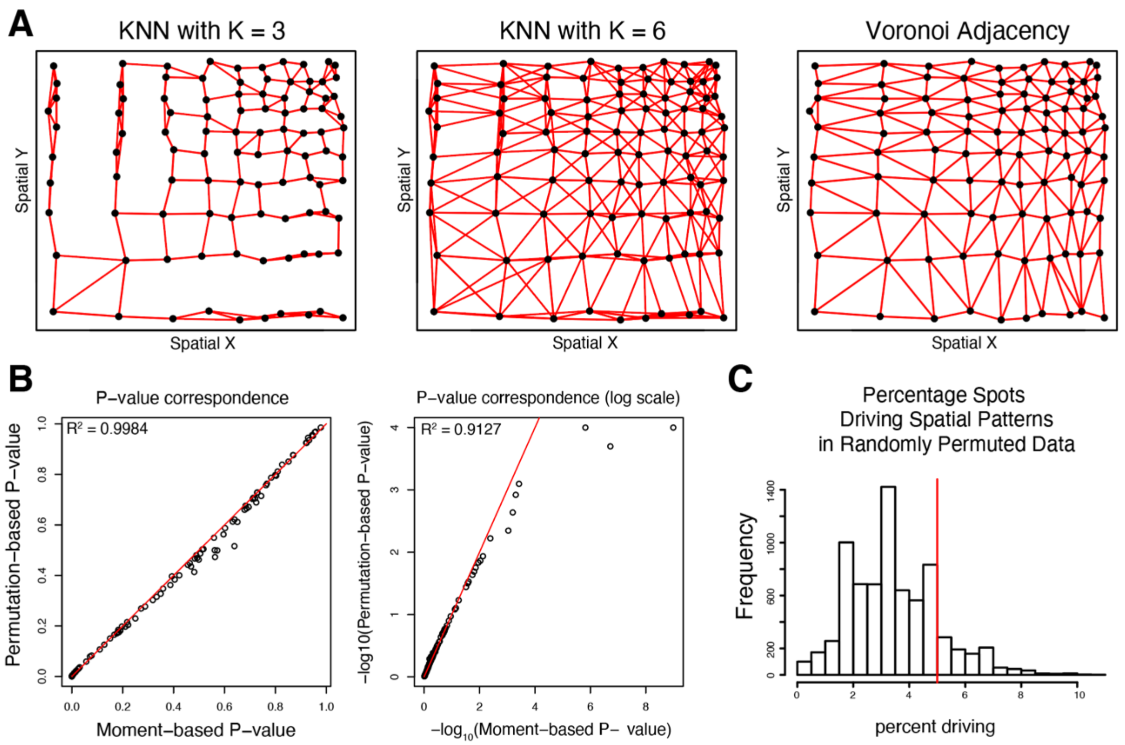

Supplemental Figure S2. Parameter and design justifications. A. Stability of Voronoi-based neighbor inference compared to *k*-nearest-neighbors-based neighbor inference. We simulate cells in space for illustrative purposes. Each point is a cell. Red lines connect the cells if they are inferred to be neighbors. *k*-nearest neighbor approach for inferring neighbors for *k*=3 (left) and *k*=6 (middle). Cells are considered neighbors if they are within the 3 spatially closest cells based on Euclidean distance. Voronoi-based approach for inferring neighbors is parameter free (right). Cells are considered neighbors if their Voronoi-tessellation share an edge. **B.** Correspondence between moment-based and permutation-based p-values in assessing significance of the Moran's I statistic. Each point is a gene. Red line denotes linear fit. P-values from the moment-based and permutation-based approach to assessing the significance of the Moran's I statistic is highly correlated on both the linear (left) and log scale (right), though log-scale p-values from the permutation-based approach for assessing the significance of the Moran's I statistic plateaus at 4 due to limitations of permutation as only 10^4 permutations were generated. **C.** Histogram of the percentage of probe spots driving spatial patterns for randomly permuted gene expression. Red line denotes 5% threshold.

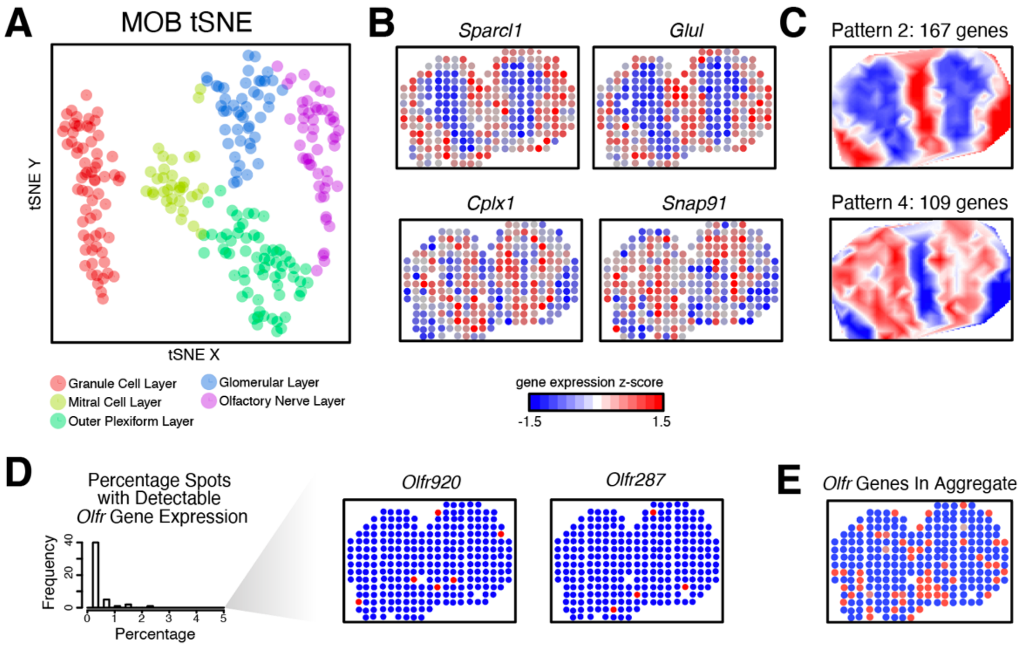

Supplemental Figure S3. Additional analysis of Spatial Transcriptomics (ST) data of the mouse olfactory bulb (MOB). A. Expression-based clustering analysis. Each point is a spot colored based on previously published annotations. The 2D tSNE embedding shows these spots to be transcriptionally distinct. **B.** Additional examples of significantly spatially heterogeneous genes. **C.** Additional examples of identified primary spatial patterns. **D.** Histogram of the percentage of spots with detectable (non-zero) *Olfr* gene expression shows most *Olfr* genes are detected in a very low percentage of spots (left). Select examples of individual *Olfr* genes are shown (right). **E.** Aggregating all *Olfr* genes by summing up their expression values enables identification of significant spatial gene expression heterogeneity (Moran's I p-value = 0.0000283).

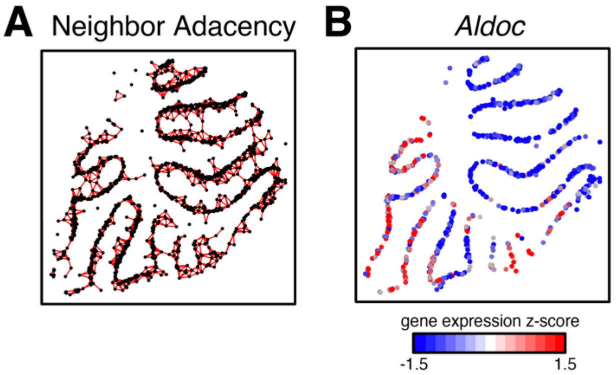

Supplemental Figure S4. Analysis of SlideSeq of the mouse cerebellum. A. Neighborhood adjacency relationship between beads that correspond to the Purkinje layer. **B.** Spatial expression pattern of one identified significantly spatially variable gene *Aldoc*.

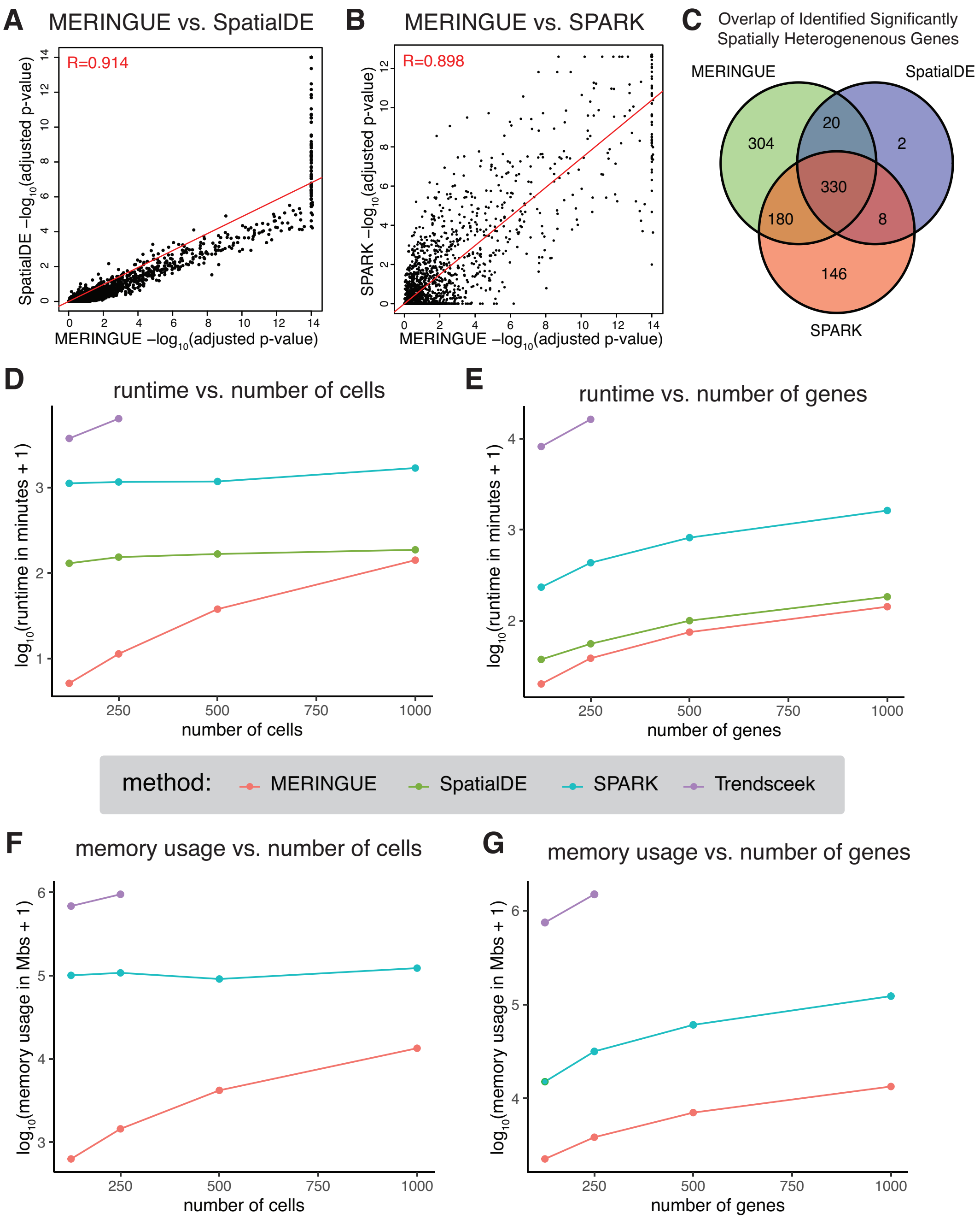

Supplemental Figure S5. Comparison of MERINGUE to previously published methods for analyzing spatial transcriptomics data using ST data of the MOB. Significance of spatial heterogeneity in terms of -$\log_{10}$(adjusted p-value) across evaluated genes for **A.** MERINGUE versus SpatialDE and **B.** MERINGUE versus SPARK. **C.** Venn diagram of genes identified as significantly spatially heterogeneous by MERINGUE, SpatialDE, and SPARK. Runtime as a function of **D**. number of cells and **E.** number of genes across spatial transcriptomics analysis methods. Memory usage, defined as the amount of memory allocated and then subsequently released after the running lines corresponding to each spatial transcriptomics analysis method in R, as a function of **F.** number of cells and **G.** number of genes.

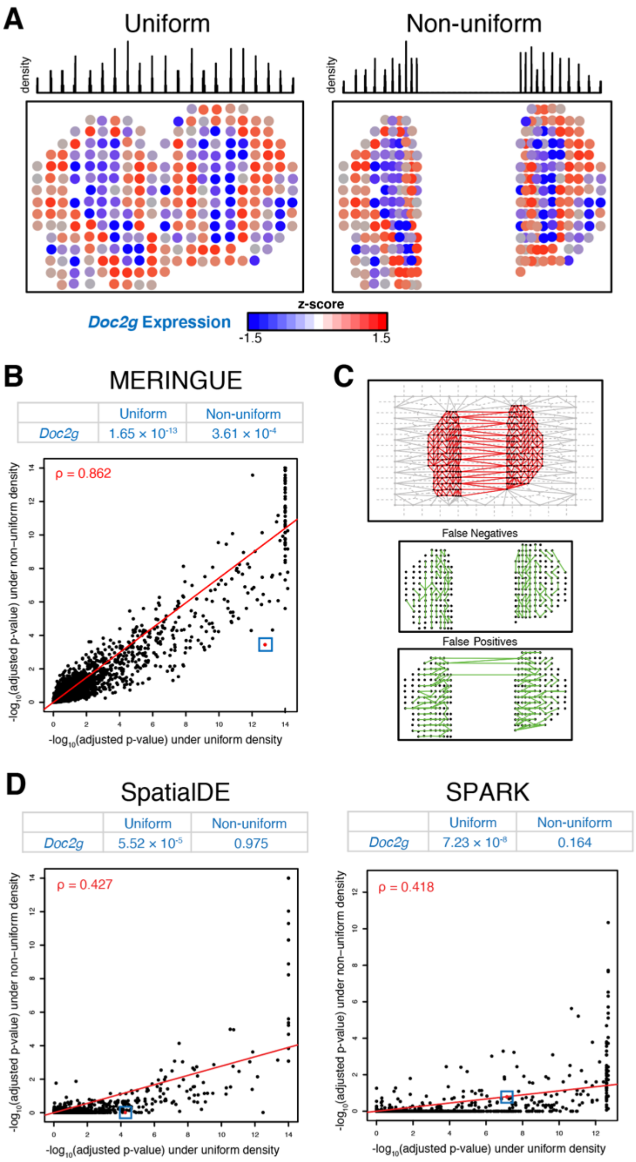

	Uniform	Non-uniform
Doc2g	1.65×10^{-13}	3.61×10^{-4}

	Uniform	Non-uniform
Doc2g	5.52×10^{-5}	0.975

	Uniform	Non-uniform
Doc2g	7.23×10^{-8}	0.164

Supplemental Figure S6. Comparison of MERINGUE to previously published methods in analyzing spatial transcriptomics data with uniform and non-uniform spot densities. A. Density distribution of probe spots under the uniform (left) and simulated non-uniform (right) density case. A sample spatially heterogeneous gene, *Doc2g*, is shown. **B.** Significance of spatial heterogeneity in terms of $-\log_{10}$(adjusted p-value) across evaluated genes under uniform and non-uniform density by MERINGUE. The $-\log_{10}$(adjusted p-value) for *Doc2g* is shown as a table and highlighted in the scatterplot as a red point with a blue square. **C.** Differences in the binary weight matrix derived from uniform versus non-uniform density. The Voronoi-based neighborhoods derived on the simulated non-uniform density spot positions (top) have missing adjacency relationships (middle) as well as new adjacency relationships (bottom) compared to neighborhoods derived on the original uniform density spot positions. **D.** Significance of spatial heterogeneity in terms of $-\log_{10}$(adjusted p-value) across evaluated genes under uniform and non-uniform density by SpatialDE and SPARK. The $-\log_{10}$(adjusted p-value) for *Doc2g* is shown as a table and highlighted in the scatterplot as a red point with a blue square.

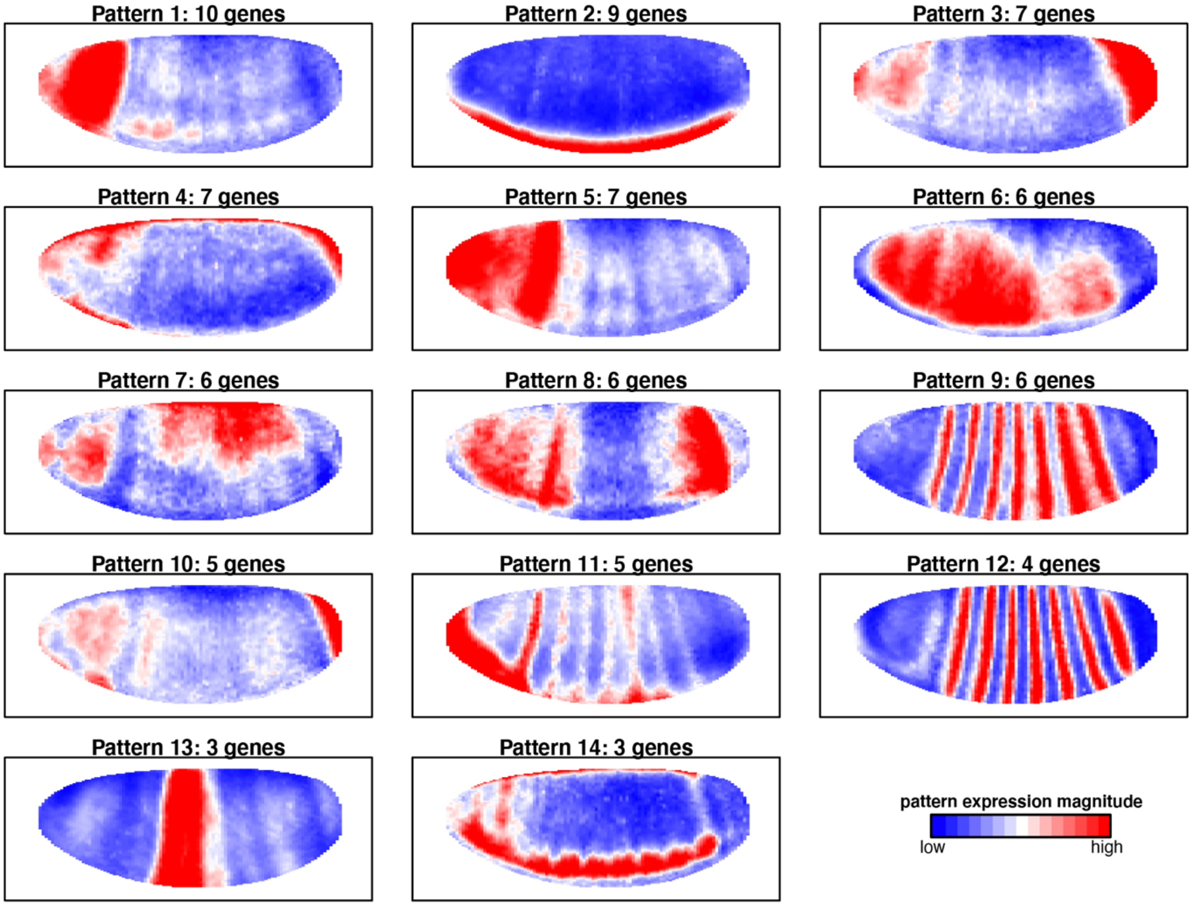

Supplemental Figure S7. Additional analysis of aligned ISH data of the *Drosophila melanogaster* embryo. Visualization of the 14 identified spatial patterns.

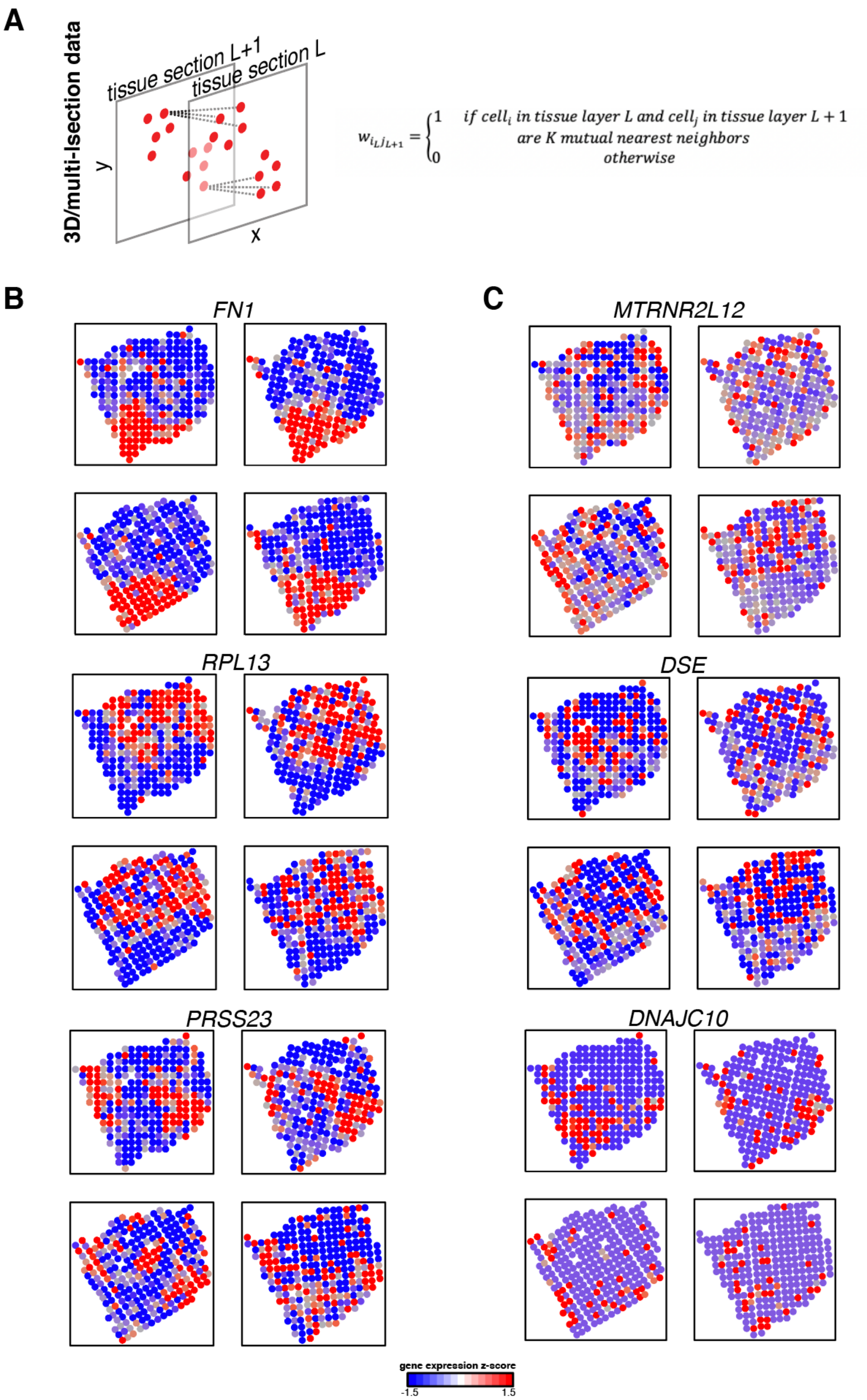

Supplemental Figure S8. Analysis of Spatial Transcriptomics data of consecutive histological sections of a breast cancer biopsy. A. To accommodate multi-layer tissue data, a binary weight matrix can be constructed by identifying mutual nearest neighbors in space between adjacent tissue layers. **B.** Examples of genes with significant spatially heterogeneous expression within and across sections. **C.** Examples of genes that exhibit significant spatially heterogeneous expression e within sections but not across sections.

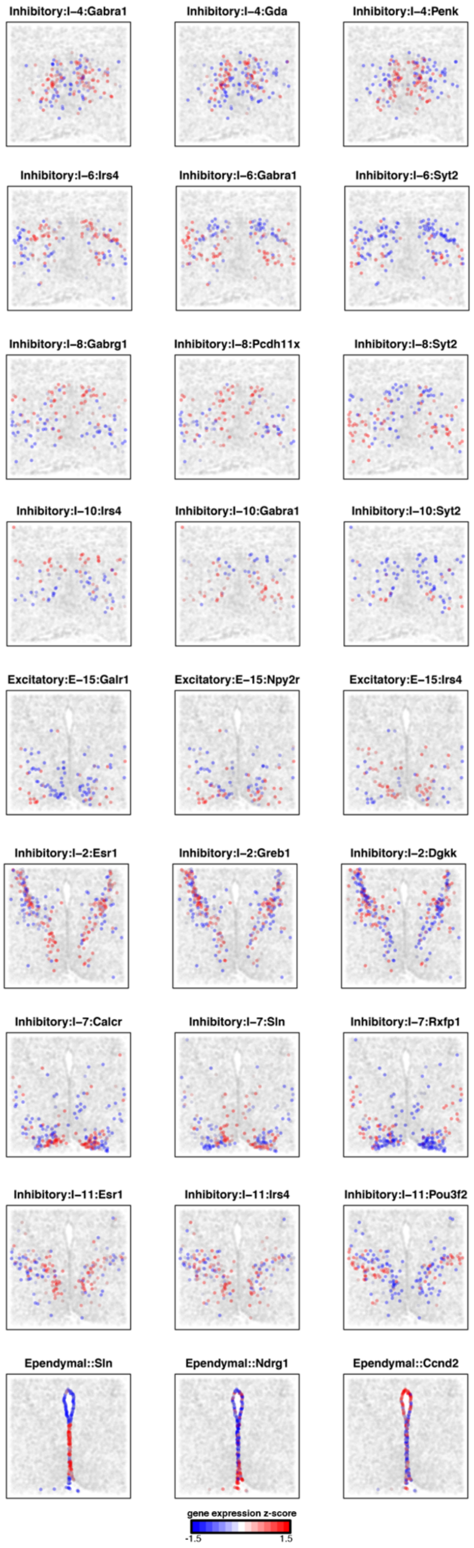

Supplemental Figure S9. Additional examples of aspects of spatial heterogeneity in the preoptic region.

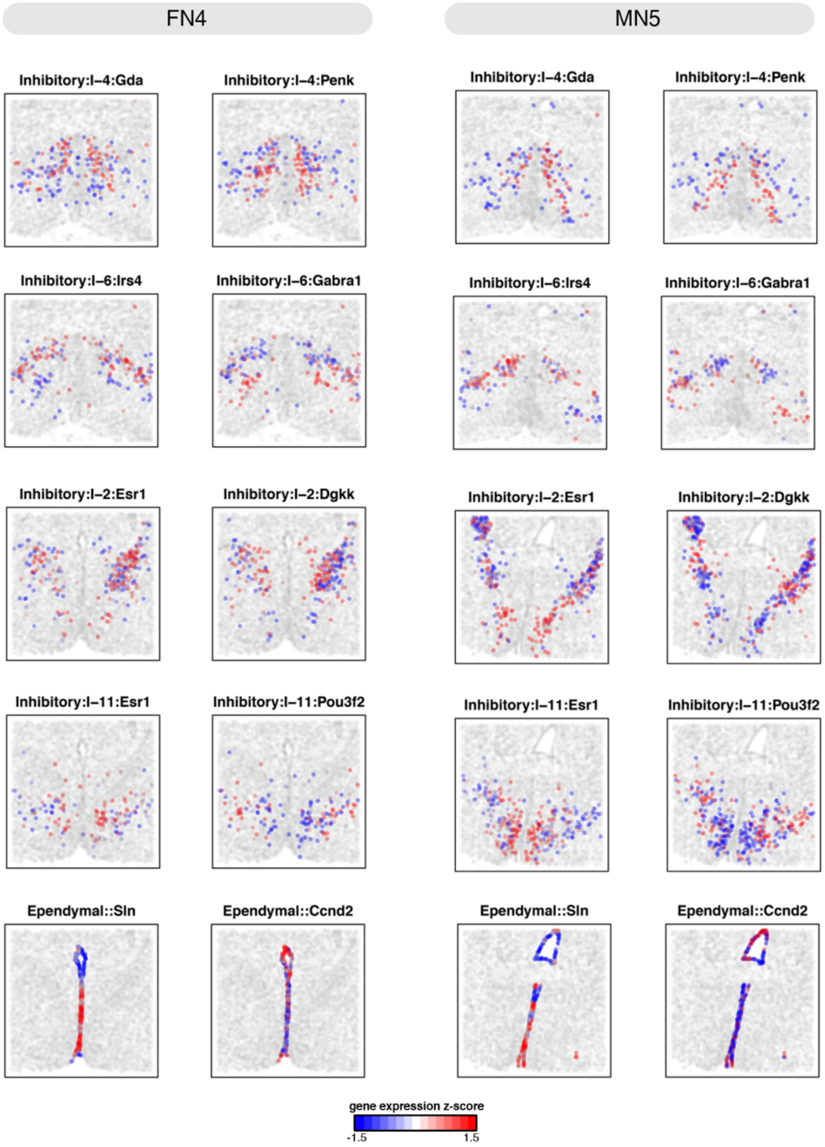

Supplemental Figure S10. Consistency of spatial patterns in the preoptic region across female and male animals. A different female (FN4) and male animal (MN5) are shown. Visual inspection shows general correspondence in spatial gene expression patterns despite minor mechanical tissue distortions and rotations.

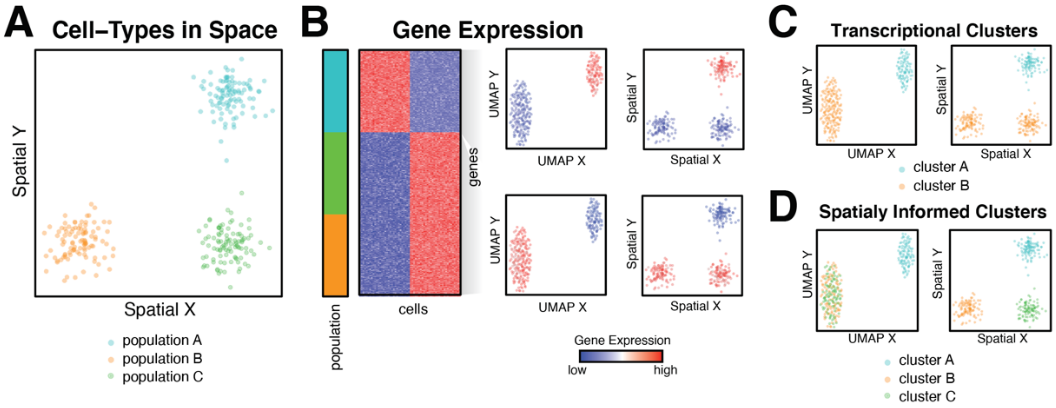

Supplemental Figure S11. Simulation of spatially informed clustering. A. 3 spatially distinct cell populations are simulated and visualized. Each point is a cell plotted in space. Cells are colored based on their simulated population. **B.** Gene expression profile of simulated cells. We simulate the population A (blue) cells to be transcriptionally distinct but the population B (orange) and C (green) cells to be transcriptionally similar. Heatmap of simulated gene expression across cell-types (left). Each row is a cell with column colors corresponding to the simulated cell-types. Red denotes high expression while blue denotes low expression. Dimensionality reduction and 2D visualization via a UMAP embedding (middle) and spatial embedding (right). Each point is a cell, colored by gene expression corresponding to the heatmap. **C.** Results of regular transcriptional clustering. Two transcriptionally distinct clusters are identified. Each cell in the UMAP embedding (left) and spatial embedding (right) are colored by the identified clusters from transcriptional clustering analysis. **D.** Results of spatially informed clustering. Three transcriptionally and spatially distinct clusters are identified. Each cell in the UMAP embedding (left) and spatial embedding (right) are colored by the identified clusters from spatially informed clustering analysis.

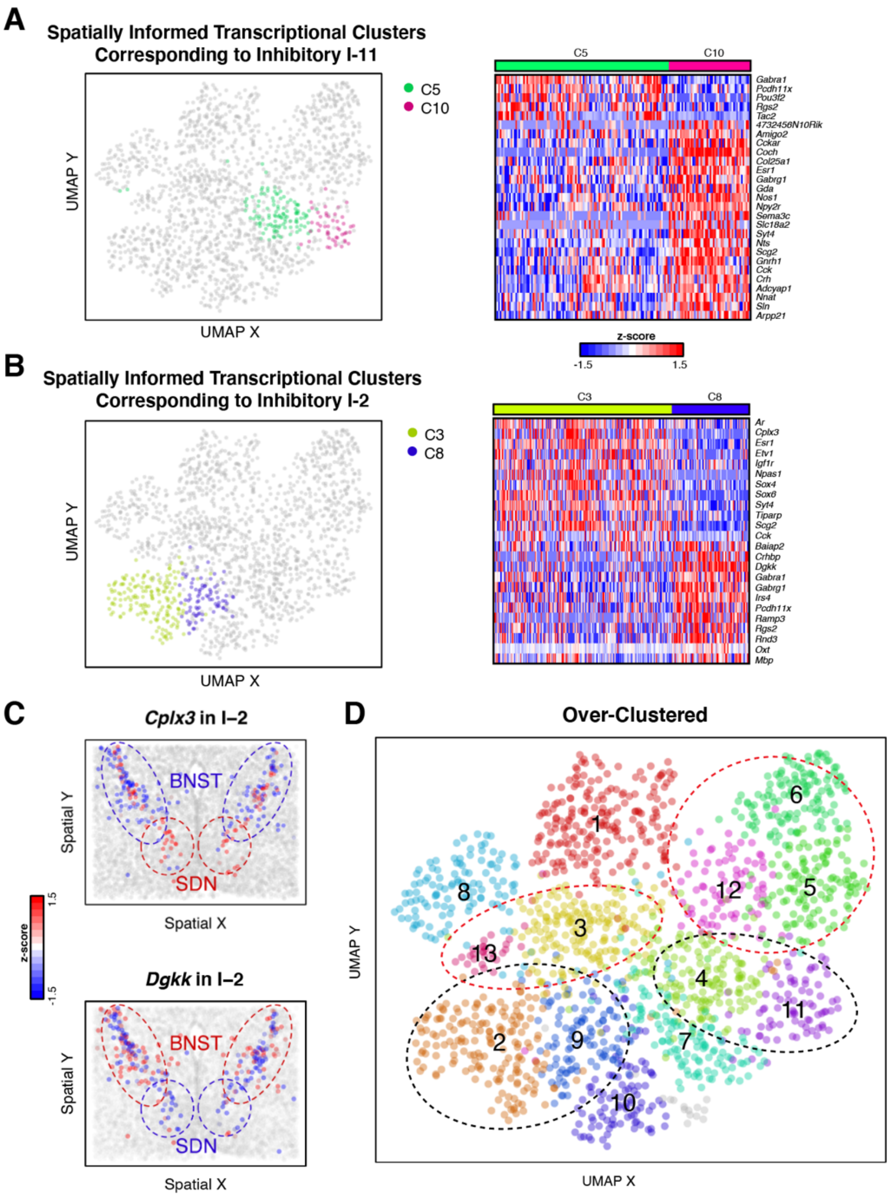

Supplemental Figure S12. Transcriptional characterization of spatially informed clusters in the preoptic region. A. UMAP embedding with spatially informed clusters C5 and C10 that corresponding to the original Inhibitory I-11 neuronal population highlighted (left). Significantly differentially expressed genes (Wilcoxon p-value < 0.05) between C5 and C10 visualized as a heatmap (right). **B.** (left) UMAP embedding with spatially informed clusters C3 and C8 that corresponding to the original Inhibitory I-2 neuronal population highlighted. Significantly differentially expressed genes (Wilcoxon p-value < 0.05) between C3 and C8 visualized as a heatmap (right). C. Expression of *Cplx3* (top) and *Dgkk* (bottom), two sample differentially expressed genes between C3 and C8, in I-2 neurons. Each point is a cell in space. I-2 neurons are colored based on their gene expression. Regions corresponding to the BNST and SDN regions are highlighted with blue and red dashed lines respectively. I-2 cells within the SDN appear to have higher *Cplx3* expression relative to I-2 cells within the BNST and conversely for *Dgkk*. **D.** Tuning regular transcriptional clustering to detect finer subtypes. I-2 and I-11, highlighted with black dashed lines, do indeed become split, though additional subtypes, highlighted with red dashed lines, appear to become oversplit.

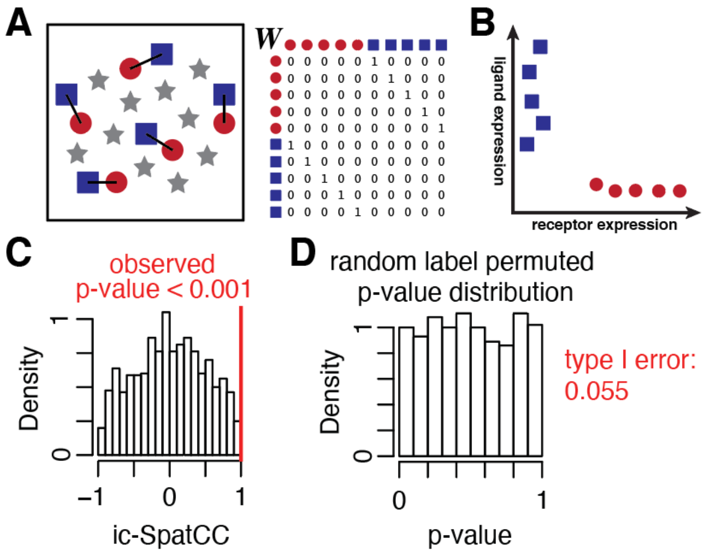

Supplemental Figure S13. Simulation of inter-cell-type spatial cross correlation. A. Three simulated cell-types in space represented as different shapes (left). We focus on communications between cell-type A (blue squares) and cell-type B (red circles). Lines are drawn between cells if they are neighbors in space and are represent both our cell-types of interest. **B.** The corresponding adjacency weight matrix W to (A). W has a value of 1 is two cells are neighbors and represent both our cell-types of interest. W has a value of 0 if two cells are not neighbors or are neighbors with the same cell-type. **C.** Simulated expression of a receptor and corresponding ligand. Receptor expression is depicted on the x-axis and ligand expression on the y-axis. Cell-type A cells express the ligand highly but not the receptor while cell-type B cells express the receptor highly but not the ligand. Correlation between the receptor and ligand gene expression across cells is therefore negative, as depicted by the negative correlation line in red. We simulate high correlation between receptor expression in cell-type B cells and ligand expression in spatially neighboring cell-type A cells. **D.** Distribution of the inter-cell-type spatial cross-correlation index (iSCI) for the ligand and receptor expression in (C) for randomly permuted cell labels provides a null distribution. The observed iSCI for the ligand and receptor expression between cell-type A and B is shown as a red horizontal line. The permutation p-value is derived by comparing the observed iSCI to the null distribution. In this case, the observed iSCI is much higher than what we expect by chance, giving it a significant p-value. **E.** Distribution of the inter-cell-type spatial cross-correlation index (iSCI) for random normally distributed gene expressions. The distribution of resulting p-values are uniformly distributed with the expected ~5% of the computed iSCI to reach significance based on a p-value < 0.05 without multiple-testing correction, suggesting a type I error rate of 5% or a 5% probability of incorrectly rejecting the true null hypothesis that the genes are not spatially cross-correlated across cell-types.

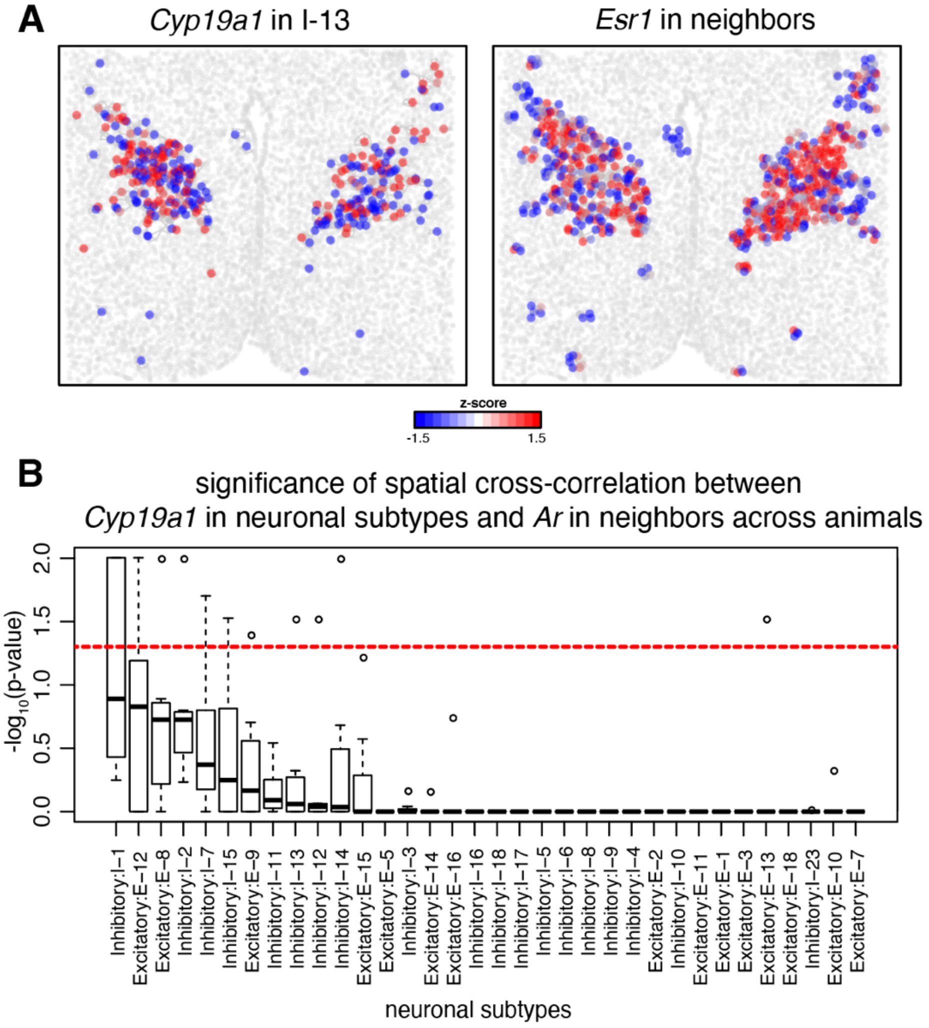

Supplemental Figure S14. Additional examples of putative cell-cell communication in the preoptic region. A. Aromatase (*Cyp19a1*) expression in I-13 neurons in one tissue layer in one animal with red indicating high expression and blue indicating low expression (left). *Esr1* expression in cells neighboring I-13 neurons in one tissue layer in one animal with red indicating high expression and blue indicating low expression (right). Representative sample shown. **B.** Distribution of -$\log_{10}$(p-values) for the spatial cross-correlation between aromatase (*Cyp19a1*) expression in neuronal subtypes and *Ar* expression in adjacent cells across animals. Red dotted line is the significance threshold.

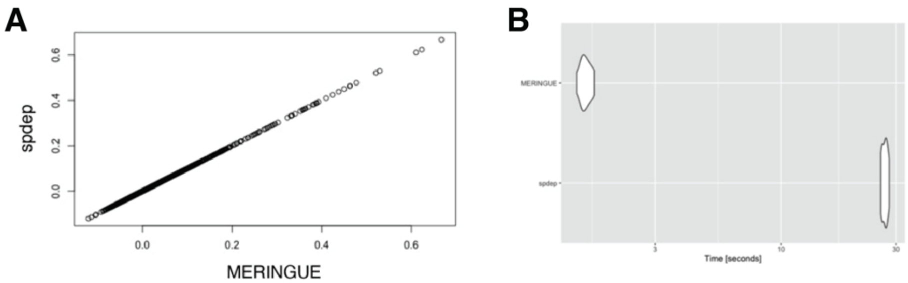

Supplemental Figure S15. Comparison of Moran's I as implemented in spdep versus MERINGUE in evaluating 2000 genes across 260 spots in the MOB dataset. A. Correlation in I statistics between spdep and MERINGUE's implementations of Moran's I (Spearman correlation R=1). **B.** Distribution of runtimes for evaluating each gene using spdep and MERINGUE's implementations of Moran's I. MERINGUE on average took 1.54 seconds to complete while spdep took 26.94 seconds per gene.

C. Supplemental References

Bivand RS, Pebesma E, Gomez-Rubio V (2013). Applied spatial data analysis with R, Second edition. Springer, NY. https://asdar-book.org/.

Fowlkes CC, Hendriks CLL, Keränen SVE, Weber GH, Rübel O, Huang M-Y, Chatoor S, DePace AH, Simirenko L, Henriquez C, et al. 2008. A quantitative spatiotemporal atlas of gene expression in the Drosophila blastoderm. *Cell* **133**: 364–74. http://www.ncbi.nlm.nih.gov/pubmed/18423206.

Johnson WE, Li C, Rabinovic A. 2007. Adjusting batch effects in microarray expression data using empirical Bayes methods. *Biostatistics* **8**: 118–27. http://www.ncbi.nlm.nih.gov/pubmed/16632515.

Karaiskos N, Wahle P, Alles J, Boltengagen A, Ayoub S, Kipar C, Kocks C, Rajewsky N, Zinzen RP. 2017. The Drosophila embryo at single-cell transcriptome resolution. *Science* **358**: 194–199. http://www.ncbi.nlm.nih.gov/pubmed/28860209

Langfelder P, Zhang B, Horvath S. 2008. Defining clusters from a hierarchical cluster tree: the Dynamic Tree Cut package for R. *Bioinformatics* **24**: 719–720. https://academic.oup.com/bioinformatics/article-lookup/doi/10.1093/bioinformatics/btm563

Moffitt JR, Bambah-Mukku D, Eichhorn SW, Vaughn E, Shekhar K, Perez JD, Rubinstein ND, Hao J, Regev A, Dulac C, et al. 2018. Molecular, spatial and functional single-cell profiling of the hypothalamic preoptic region. *Science* eaau5324. http://www.ncbi.nlm.nih.gov/pubmed/30385464

Ramilowski JA, Goldberg T, Harshbarger J, Kloppman E, Lizio M, Satagopam VP, Itoh M, Kawaji H, Carninci P, Rost B, et al. 2015. A draft network of ligand-receptor-mediated multicellular signalling in human. *Nat Commun* **6**.

Rodriques SG, Stickels RR, Goeva A, Martin CA, Murray E, Vanderburg CR, Welch J, Chen LM, Chen F, Macosko EZ. 2019. Slide-seq: A scalable technology for measuring genome-wide expression at high spatial resolution. *Science* **363**: 1463–1467. http://www.ncbi.nlm.nih.gov/pubmed/30923225

Ståhl PL, Salmén F, Vickovic S, Lundmark A, Navarro JF, Magnusson J, Giacomello S, Asp M, Westholm JO, Huss M, et al. 2016. Visualization and analysis of gene expression in tissue sections by spatial transcriptomics. *Science* **353**: 78–82. http://www.ncbi.nlm.nih.gov/pubmed/27365449

Sun S, Zhu J, Zhou X. 2020. Statistical analysis of spatial expression patterns for spatially resolved transcriptomic studies. *Nat Methods* **17**: 193–200. /pmc/articles/PMC7233129/?report=abstract
