# Supplementary material for: Characterizing spatial gene expression heterogeneity in spatially resolved single-cell transcriptomic data with nonuniform cellular densities
Source: Genome Res. 2021 Oct;31(10):1843–55. doi: 10.1101/gr.271288.120 (PMC8494224; doi:10.1101/gr.271288.120)
Supplement: Supplemental Material [file supp_gr.271288.120_Supplemental_Software_MERINGUE_1.0.tar.gz › MERINGUE/inst/doc/BCL_analysis.html]

Multi-section 3D Breast Cancer Spatial Transcriptomics Analysis with MERINGUE


# Multi-section 3D Breast Cancer Spatial Transcriptomics Analysis with MERINGUE

#### Jean Fan

In this vignette, we will walk through an analysis of spatial transcriptomics data for multiple consecutive tissue slices of a breast cancer biopsy. Briefly, for spatial transcriptomics, histological sections are placed on a grid of poly(dT) probe spots approximately 100 um in diameter, each with a unique DNA barcode. By resolving the DNA barcodes, spatial transcriptomics enables matching of detected mRNA abundances with their original spatially resolved spot, resulting in full transcriptome RNA-sequencing data with homogenously-spaced two-dimensional positional information at the pixel level. See the original publication for more information.

The data has been prepared for you and is available as a part of the package. Here, each dataset is a list containing two items: `pos` is a dataframe where each row is a probe spot’s x and y positions in space, and `counts` is a counts matrix where each column is a probe spot and each row is a gene.

Although a total of 4 tissue sections are available, we will focus on analyzing 3 tissue sections for demonstrative purposes.

```
suppressMessages(library(MERINGUE))

data(BCL) # object contains counts and positions for all 4 sections. Will only need sections 1-3
head(BCL$pos)
BCL$counts[1:5,1:5]

## parse out the 3 sections
BCL1.pos <- BCL$pos[BCL$pos$slice==1,][,c("x", "y")]
BCL2.pos <- BCL$pos[BCL$pos$slice==2,][,c("x", "y")]
BCL3.pos <- BCL$pos[BCL$pos$slice==3,][,c("x", "y")]
BCL1.counts <- BCL$counts[,BCL$pos$slice==1]
BCL2.counts <- BCL$counts[,BCL$pos$slice==2]
BCL3.counts <- BCL$counts[,BCL$pos$slice==3]
```

```
##                                x          y slice
## L1-ACAACTATGGGTTGGCGG -0.5602323 -0.8045787     1
## L1-ACATCACCTGCGCGCTCT -3.3702323  4.1954213     1
## L1-ACATGTGTCGTTTCAAGA  0.5217677 -2.7195787     1
## L1-ACATTTAAGGCGCATGAT -2.4342323  1.1894213     1
## L1-ACCACTGTAATCTCCCAT  7.6007677 -3.7575787     1
## L1-ACCCGGCGTAACTAGATA -0.4612323 -4.7785787     1
## 5 x 5 sparse Matrix of class "dgCMatrix"
##        L1-ACAACTATGGGTTGGCGG L1-ACATCACCTGCGCGCTCT L1-ACATGTGTCGTTTCAAGA
## A1BG                       .                     .                     .
## A2M                        .                     2                     6
## A2ML1                      .                     .                     .
## A4GALT                     .                     .                     .
## AAAS                       .                     .                     .
##        L1-ACATTTAAGGCGCATGAT L1-ACCACTGTAATCTCCCAT
## A1BG                       .                     .
## A2M                        2                     3
## A2ML1                      .                     .
## A4GALT                     .                     3
## AAAS                       .                     .
```

We will first combine the data from all three samples into a single counts matrix. We will also combine the position information into a list of positions.

```
# Get common set of genes for the sections
genes.have <- Reduce(intersect, list(
  rownames(BCL1.counts),
  rownames(BCL2.counts),
  rownames(BCL3.counts)
))

# Combine into large counts matrix. Counts of genes for all the sections being assessed
counts <- cbind(
  BCL1.counts[genes.have,],
  BCL2.counts[genes.have,],
  BCL3.counts[genes.have,]
)

# section factor
section <-  c(
  rep('L1', ncol(BCL1.counts)),
  rep('L2', ncol(BCL2.counts)),
  rep('L3', ncol(BCL3.counts))
)
names(section) <- colnames(counts)

# List of positions
posList <- list(
  BCL1.pos[colnames(BCL1.counts),],
  BCL2.pos[colnames(BCL2.counts),],
  BCL3.pos[colnames(BCL3.counts),]
)
```

We will then filter out genes with fewer than 100 counts across all probe spots and filter out probe spots with fewer than 100 total counts and update our position information accordingly.

```
cc <- cleanCounts(counts, min.reads = 100, min.lib.size = 100, plot=TRUE)
```

```
mat <- normalizeCounts(cc, log=FALSE, verbose=TRUE)
```

```
## Normalizing matrix with 767 cells and 4985 genes.
```

```
## normFactor not provided. Normalizing by library size.
```

```
## Using depthScale 1e+06
```

```
posList[[1]] <- posList[[1]][intersect(rownames(posList[[1]]), colnames(mat)),]
posList[[2]] <- posList[[2]][intersect(rownames(posList[[2]]), colnames(mat)),]
posList[[3]] <- posList[[3]][intersect(rownames(posList[[3]]), colnames(mat)),]
```

Now, let’s visualize the probe spots for each section. For demonstrative purposes, we will look for evidence of significant spatial aggregation for 1000 randomly chosen genes.

```
# Plot
par(mfrow=c(1,3), mar=rep(2,4))
plotEmbedding(posList[[1]], groups=section, main='section 1', cex=2)
plotEmbedding(posList[[2]], groups=section, main='section 2', cex=2)
plotEmbedding(posList[[3]], groups=section, main='section 3', cex=2)
```

```
# Sample 1000 genes for demonstrative purposes only
set.seed(0)
test <- sample(rownames(mat), 1000)
mat <- mat[test,]
```

# Individual section analysis

Now, we can analyze each section separately to look for significantly spatially aggregated genes. For each section, we will construct a binary weight matrix that represents the spatial adjacency relationship between probe spots. We will then look for significantly spatially aggregated genes driven by more than 5% of probe spots.

```
helper <- function(pos, mat) {
  w <- getSpatialNeighbors(pos, filterDist = 3)
  plotNetwork(pos, w)
  # get spatially clustered genes
  I <- getSpatialPatterns(mat, w)
  # filter for significant hits driven by more than 10% of cells
  results.filter <- filterSpatialPatterns(mat = mat,
                                          I = I,
                                          w = w,
                                          adjustPv = TRUE,
                                          alpha = 0.05,
                                          minPercentCells = 0.05,
                                          verbose = TRUE)
  # return results
  list(I=I, sig.genes=results.filter)
}

# Analyze each section using helper function
par(mfrow=c(1,3), mar=rep(2,4))
L1 <- helper(posList[[1]], mat[, rownames(posList[[1]])])
```

```
## Number of significantly autocorrelated genes: 92
```

```
## ...driven by > 12.7 cells: 48
```

```
L2 <- helper(posList[[2]], mat[, rownames(posList[[2]])])
```

```
## Number of significantly autocorrelated genes: 87
```

```
## ...driven by > 12.5 cells: 44
```

```
L3 <- helper(posList[[3]], mat[, rownames(posList[[3]])])
```

```
## Number of significantly autocorrelated genes: 67
```

```
## ...driven by > 13.15 cells: 29
```

# Multi-section analysis

We can also specifically look for spatially aggregated genes that with patterns that are consistent across sections. To do this, we will create a new binary weight matrix that specially connects probe spots across adjacent tissue sections. Two probe spots will have a weight of 1 if they are within the k mutual nearest neighbors in space across two sections. We can then use this new binary weight matrix to identify spatially aggregated genes that are aggregated across sections.

```
# K-mutual nearest neighbors across sections
cw <- getCrossLayerNeighbors(posList, k=3)
# Look for genes exhibiting spatial autocorrelation across layers
I <- getSpatialPatterns(mat, cw)
results.filter <- filterSpatialPatterns(mat = mat,
                                        I = I,
                                        w = cw,
                                        adjustPv = TRUE,
                                        alpha = 0.05,
                                        minPercentCells = 0.05/4,
                                        verbose = TRUE)
```

```
## Number of significantly autocorrelated genes: 85
```

```
## ...driven by > 9.5875 cells: 79
```

```
cross <- list(I=I, sig.genes=results.filter)
```

We can now compare our results from our individual sample analyses and our cross sample analyses. Let’s look at a few genes that are significantly aggregated within multiple sections AND consistent across sections.

```
gdups <- unlist(list('L1'=L1$sig.genes, 
                     'L2'=L2$sig.genes, 
                     'L3'=L3$sig.genes
))
## genes that are significantly spatially variable in multiple sections
gdups <- gdups[duplicated(gdups)]
## genes that are significantly spatially variable across sections
gcross <- cross$sig.genes
## both 
gall <- intersect(gcross, gdups)
## order by Moran's I
gall <- gall[order(L1$I[gall,]$observed, decreasing=TRUE)]

# Plot few consistent genes
invisible(lapply(gall[1:2], function(g1) {
  par(mfrow=c(1,3), mar=rep(2,4))
  plotEmbedding(posList[[1]], 
                colors=winsorize(scale(mat[g1, rownames(posList[[1]])])[,1]), 
                main=paste0(g1, '\n section 1'),
                cex=2, verbose=FALSE)
  plotEmbedding(posList[[2]], 
                colors=winsorize(scale(mat[g1, rownames(posList[[2]])])[,1]), 
                main=paste0(g1, '\n section 2'), 
                cex=2, verbose=FALSE)
  plotEmbedding(posList[[3]], 
                colors=winsorize(scale(mat[g1, rownames(posList[[3]])])[,1]), 
                main=paste0(g1, '\n section 3'), 
                cex=2, verbose=FALSE)
}))
```

Alternatively, we can look at genes that are spatially aggregated within multiple sections but not in a manner that is consistent across sections. Such inconsistent patterns may be indicative of section-specific structures or technical artefacts.

```
gdiff <- setdiff(gdups, gcross)
gdiff <- gdiff[order(L1$I[gdiff,]$observed, decreasing=TRUE)]

# Plot inconsistent gene
invisible(lapply(gdiff[1], function(g1) {
  par(mfrow=c(1,3), mar=rep(2,4))
  plotEmbedding(posList[[1]], 
                colors=winsorize(scale(mat[g1, rownames(posList[[1]])])[,1]), 
                main=paste0(g1, '\n section 1'),
                cex=2, verbose=FALSE)
  plotEmbedding(posList[[2]], 
                colors=winsorize(scale(mat[g1, rownames(posList[[2]])])[,1]), 
                main=paste0(g1, '\n section 2'), 
                cex=2, verbose=FALSE)
  plotEmbedding(posList[[3]], 
                colors=winsorize(scale(mat[g1, rownames(posList[[3]])])[,1]), 
                main=paste0(g1, '\n section 3'), 
                cex=2, verbose=FALSE)
}))
```

# Additional exercises

1. Integrate the 4th tissue section.
2. What primary spatial patterns are marked by genes consistent across sections?
3. What primary spatial patterns are marked by genes inconsistent across sections?
