# Supplementary material for: Characterizing spatial gene expression heterogeneity in spatially resolved single-cell transcriptomic data with nonuniform cellular densities
Source: Genome Res. 2021 Oct;31(10):1843–55. doi: 10.1101/gr.271288.120 (PMC8494224; doi:10.1101/gr.271288.120)
Supplement: Supplemental Material [file supp_gr.271288.120_Supplemental_Software_MERINGUE_1.0.tar.gz › MERINGUE/inst/doc/drosophila_3D_analysis.html]

3D Drosophila Spatial Transcriptomics Analysis with MERINGUE


# 3D Drosophila Spatial Transcriptomics Analysis with MERINGUE

#### Jean Fan

```
library(MERINGUE)
```

In this vignette, we will walk through an analysis of spatial gene expression data for the drosophila embryo from the Berkeley Drosophila Transcription Network Project. Notably, 3D spatial coordinates are available for this dataset, allowing us to analyze beyond a single plane or tissue section.

```
data("drosophila")
pos <- drosophila$pos
gexp <- drosophila$mat

par(mfrow=c(1,3), mar=rep(2,4))
g <- 'apt'
plotEmbedding(pos[,c(1,2)], col=gexp[g,], main='X-Y')
plotEmbedding(pos[,c(3,2)], col=gexp[g,], main='Z-Y')
plotEmbedding(pos[,c(1,3)], col=gexp[g,], main='X-Z')
```

We can consider the 3D coordinates in our construction of neighbor relationships.

```
N <- getSpatialNeighbors(pos, filterDist = 10, verbose=TRUE)
```

```
## Filtering by distance: 10...
```

```
## Binarizing adjacency weight matrix ...
```

```
## Done!
```

Optionally, this 3D neighbor network can be visualized using RGL, a 3D real-time rendering system for R. WARNING: there will be approximately 25,500 edges to draw and thus will take a significant amount of time if rendered to your screen (for example using X11). We recommend drawing to file.

```
## plot
require(rgl)
MERINGUE:::plotNetwork3D(pos, N, size=1)
## save plot
rgl.snapshot(paste0(dir(), 'neighbors_3D-flat.png'))
```

In this particular case, all 84 genes were chosen for their known spatial patterning. We can double check that they all indeed show up as significantly spatially variable.

```
## analyze
results <- getSpatialPatterns(gexp, N)
filter <- filterSpatialPatterns(mat = gexp, 
                                I = results, 
                                w = N,
                                alpha = 0.05,
                                details = TRUE, 
                                minPercentCells = 0.05)
```

```
## Number of significantly autocorrelated genes: 84
```

```
## ...driven by > 151.75 cells: 84
```

```
print(head(filter))
```

```
##          observed      expected          sd p.value p.adj minPercentCells
## aay     0.9093090 -0.0003295979 0.008910810       0     0      0.20790774
## Ama     0.9579342 -0.0003295979 0.008933794       0     0      0.53476112
## Ance    0.8862914 -0.0003295979 0.008894401       0     0      0.07710049
## Antp    0.8450446 -0.0003295979 0.008918188       0     0      0.19868204
## apt     0.9343848 -0.0003295979 0.008930957       0     0      0.31070840
## Blimp.1 0.8375291 -0.0003295979 0.008932513       0     0      0.40988468
```

To identify spatial patterns, we can restrict our analysis to a subset of pair-rule genes for demo purposes.

```
par(mfrow=c(2,4), mar=rep(2,4))
## pair-rule genes
sub <- c('eve', 'prd', 'odd', 'run', 'ftz', 'trn', 'h', 'dpn')
invisible(lapply(sub, function(g) {
  plotEmbedding(pos[,c(1,3)], 
                col=gexp[g,], 
                main=g)
}))
```

We can use our spatial cross correlation analysis to group genes into their primary spatial patterns.

```
scc <- spatialCrossCorMatrix(gexp[sub,], N)
method = 'ward.D'
par(mfrow=c(1,2), mar=rep(2,4))
ggroup <- groupSigSpatialPatterns(pos[,c(1,3)], gexp, scc,
                                    hclustMethod = method,
                                    deepSplit = 2,
                                    binSize = 50,
                                    power = 1)
```

```
## Patterns detected:
```

```
##  ..cutHeight not given, setting it to 1.71  ===>  99% of the (truncated) height range in dendro.
##  ..done.
## groups
## 1 2 
## 4 4
```

Indeed, we are able to distinguish 2 patterns corresponding to even and odd pair-rule genes.

```
# Look at pattern association
gcol <- rainbow(length(levels(ggroup$groups)), v=0.5)[ggroup$groups]
names(gcol) <- names(ggroup$groups)
heatmap(scc[ggroup$hc$labels, ggroup$hc$labels], scale='none', 
        Colv=as.dendrogram(ggroup$hc), 
        Rowv=as.dendrogram(ggroup$hc), 
        labCol=NA,
        RowSideColors=gcol[ggroup$hc$labels],
        ColSideColors=gcol[ggroup$hc$labels],
        col=colorRampPalette(c('white', 'black'))(100)
)
```

# Additional exercises

1. Analyze all 84 genes.
2. Instead of analyzing the 3D spatial coordinates, analyze the 2D X-Z projection. How are detected spatial patterns similar or different?
