# Supplementary material for: Characterizing spatial gene expression heterogeneity in spatially resolved single-cell transcriptomic data with nonuniform cellular densities
Source: Genome Res. 2021 Oct;31(10):1843–55. doi: 10.1101/gr.271288.120 (PMC8494224; doi:10.1101/gr.271288.120)
Supplement: Supplemental Material [file supp_gr.271288.120_Supplemental_Software_MERINGUE_1.0.tar.gz › MERINGUE/inst/doc/mOB_analysis.html]

mOB Spatial Transcriptomics Analysis with MERINGUE


# mOB Spatial Transcriptomics Analysis with MERINGUE

#### Jean Fan

In this vignette, we will walk through an analysis of spatial transcriptomics data for the mouse olfactory bulb (mOB). Briefly, for spatial transcriptomics, histological sections are placed on a grid of poly(dT) probe spots approximately 100 um in diameter, each with a unique DNA barcode. By resolving the DNA barcodes, spatial transcriptomics enables matching of detected mRNA abundances with their original spatially resolved spot, resulting in full transcriptome RNA-sequencing data with homogenously-spaced two-dimensional positional information at multi-cellular spot resolution. See the original publication for more information.

The mOB data has been prepared for you and is available as a part of the package. Here, `pos` is a dataframe where each row is a probe spot’s x and y positions in space, and `cd` is a counts matrix where each column is a probe spot and each row is a gene.

```
suppressMessages(library(MERINGUE))
data(mOB)
pos <- mOB$pos
cd <- mOB$counts
```

First, we will filter out poor spots, defined as those with fewer than 100 counts. Likewise, we will filter out poor genes, defined as those with fewer than 100 counts. We will then normalize to counts per million (CPM). An appropriate normalization will be crucial to ensure that our later identified spatial patterns are not driven by technical artifacts such as library size differences.

```
# Remove poor datasets and genes
counts <- cleanCounts(counts = cd, 
                      min.reads = 100, 
                      min.lib.size = 100, 
                      plot=TRUE,
                      verbose=TRUE)
```

```
## Converting to sparse matrix ...
```

```
## Filtering matrix with 262 cells and 15928 genes ...
```

```
## Resulting matrix has 260 cells and 7365 genes
```

```
pos <- pos[colnames(counts),]

# CPM normalize
mat <- normalizeCounts(counts = counts, 
                       log=FALSE,
                       verbose=TRUE)
```

```
## Normalizing matrix with 260 cells and 7365 genes.
```

```
## normFactor not provided. Normalizing by library size.
```

```
## Using depthScale 1e+06
```

# Spatially-unaware analysis

To better understand the value of integrating spatial information, we will first perform a spatially-unaware analysis. Without considering the spatial information of each probe spot, we will simply perform dimensionality reduction and graph-based clustering to identify transcriptional subpoplations in the mOB. Note for spatial transcriptomics, a transcriptional subpopulation in this context may reflect underlying cell-type transcriptional differences or cell-type composition differences among probe spots. In this particular instance, the identified transcriptional subpopulations primarily reflect underlying cell-type. We can annotate the identified clusters based on their proposed cell-type in accordance with the original publication. We can then visualize the data using a tSNE embedding.

```
# Dimensionality reduction by PCA on log10 CPM expression values
pcs.info <- prcomp(t(log10(as.matrix(mat)+1)), center=TRUE)
nPcs <- 5
pcs <- pcs.info$x[,1:nPcs]

# 2D embedding by tSNE
emb <- Rtsne::Rtsne(pcs,
             is_distance=FALSE,
             perplexity=30,
             num_threads=1,
             verbose=FALSE)$Y
rownames(emb) <- rownames(pcs)

# Graph-based cluster detection
k <- 30
com <- getClusters(pcs, k, weight=TRUE)

# Manually annotate identified clusters with cell-types
annot <- as.character(com); names(annot) <- names(com)
annot[com==4] <- '1: Granule Cell Layer'
annot[com==1] <- '2: Mitral Cell Layer'
annot[com==3] <- '3: Outer Plexiform Layer'
annot[com==2] <- '4: Glomerular Layer'
annot[com==5] <- '5: Olfactory Nerve Layer'
annot <- as.factor(annot)

# Plot
par(mfrow=c(1,2), mar=rep(1,4))
plotEmbedding(emb, groups=annot, 
              show.legend=TRUE, xlab=NA, ylab=NA,
              verbose=FALSE)
plotEmbedding(pos, groups=annot, 
              cex=1, xlab=NA, ylab=NA,
              verbose=FALSE)
```

Having identified multiple transcriptionally distinct clusters of spots that may represent distinct cell-types, we may be interested in identifying marker genes for each cell-type. We can use a Wilcox rank-test to look for genes that are significantly upregulated in each cell-type compared to all others. For demonstration purposes, we will restrict analysis to 2000 random genes.

```
# Sample 2000 genes for demo purposes only to minimize runtime for demo only
set.seed(0)
test <- sample(rownames(mat), 2000)

# Identify significantly differentially upregulated genes
# in each identified cluster by Wilcox test
dg <- getDifferentialGenes(as.matrix(mat[test,]), annot)
dg.sig <- lapply(dg, function(x) {
  x <- x[x$p.adj < 0.05,]
  x <- na.omit(x)
  x <- x[x$highest,]
  rownames(x)
})
print(lapply(dg.sig, length))
```

```
## [1] "Running differential expression with 5 clusters ... "
## [1] "Summarizing results ... "
## $`1: Granule Cell Layer`
## [1] 437
## 
## $`2: Mitral Cell Layer`
## [1] 109
## 
## $`3: Outer Plexiform Layer`
## [1] 4
## 
## $`4: Glomerular Layer`
## [1] 67
## 
## $`5: Olfactory Nerve Layer`
## [1] 38
```

Indeed, we are able to identify a number of marker genes per cell-type. We can visualize the results as a heatmap, where each column is a spot with column colors denoting the cell-types, and each row is a gene with row colors denoting which subpopulation it is a marker for.

```
dg.genes <- unlist(dg.sig)
ggroup <- unlist(lapply(1:length(dg.sig), function(i) { 
  rep(names(dg.sig)[i], length(dg.sig[[i]]))
}))
names(ggroup) <- dg.genes
ggroup <- factor(ggroup)

# Plot
ccol <- rainbow(length(levels(annot)))[annot]
names(ccol) <- names(annot) # column colors
gcol <- rainbow(length(levels(ggroup)), v=0.5)[ggroup]
names(gcol) <- names(ggroup) # row colors

m <- as.matrix(mat[dg.genes, names(sort(annot))])
m <- winsorize(t(scale(t(m))))
heatmap(m, scale="none", 
          Colv=NA, Rowv=NA, labRow=NA, labCol=NA,
          ColSideColors=ccol[colnames(m)],
          RowSideColors=gcol[rownames(m)],
          col=colorRampPalette(c('blue', 'white', 'red'))(100)
)
```

Because transcriptionally distinct cell-types are spatially organized in the mOB, we expect a spatially-aware approach to pick up similar spatial patterns and spatially variable genes.

# Spatially-aware analysis

So now, to take an orthologonal spatially-aware approach, we will apply `MERINGUE` to identify spatially aggregated genes and group them into primary spatial patterns. To integrate the spatial information, we will create an adjacency weight matrix. We will use a binary weighting scheme here, where two probe spots will be connected with a weight of 1 if they are neighbors with each other, and otherwise not connected with a weight of 0.

```
# Get neighbor-relationships
w <- getSpatialNeighbors(pos, filterDist = 2.5)
plotNetwork(pos, w)
```

We will then use this adjacency weight matrix to assess the same set of 2000 genes for evidence of statistically significant spatial auto-correlation or spatial aggregation.

```
# Identify sigificantly spatially auto-correlated genes
start_time <- Sys.time()
I <- getSpatialPatterns(mat[test,], w)
end_time <- Sys.time()
print(end_time - start_time)
```

```
## Time difference of 1.296388 secs
```

We will further correct for multiple-testing, use an adjusted p-value threshold of 0.05, and restrict to genes driven by more than 5% of probe spots to further reduce false positives.

```
results.filter <- filterSpatialPatterns(mat = mat[test,],
                                        I = I,
                                        w = w,
                                        adjustPv = TRUE,
                                        alpha = 0.05,
                                        minPercentCells = 0.05,
                                        verbose = TRUE)
```

```
## Number of significantly autocorrelated genes: 256
```

```
## ...driven by > 13 cells: 230
```

Indeed, we identify a number of spatially aggregated genes. We anticipate that these spatially aggregated genes will likely represent a more limited number of primary spatial patterns. This could be due to their inherent co-expression within cell-types or for other biological reasons. Therefore, we can compute a spatial cross-correlation for all gene pairs.

```
# Compute spatial cross correlation matrix
scc <- spatialCrossCorMatrix(mat = as.matrix(mat[results.filter,]), 
                             weight = w)
```

We will then apply hierarchical clustering using the `ward.D` linkage criteria along with dynamic tree cutting with a `deepSplit` tuning parameter set to 2. Genes that are spatially co-localized will be grouped into the same spatial pattern.

```
# Identify primary patterns
par(mfrow=c(2,2), mar=rep(2,4))
ggroup <- groupSigSpatialPatterns(pos = pos, 
                                  mat = as.matrix(mat[results.filter,]), 
                                  scc = scc, 
                                  power = 1, 
                                  hclustMethod = 'ward.D', 
                                  deepSplit = 2,
                                  zlim=c(-1.5,1.5))
```

```
## Patterns detected:
```

```
##  ..cutHeight not given, setting it to 19.6  ===>  99% of the (truncated) height range in dendro.
##  ..done.
## groups
##  1  2  3 
## 88 88 54
```

We can visualize the spatial cross-correlation matrix to ensure that our pattern grouping is reasonable. Alternative linkage criteria for hierarchical clustering and `deepSplit` values in dynamic tree cutting may be used.

```
# Look at pattern association
gcol <- rainbow(length(levels(ggroup$groups)), v=0.5)[ggroup$groups]
names(gcol) <- names(ggroup$groups)
heatmap(scc[ggroup$hc$labels, ggroup$hc$labels], scale='none', 
        Colv=as.dendrogram(ggroup$hc), 
        Rowv=as.dendrogram(ggroup$hc), 
        labRow=NA, labCol=NA,
        ColSideColors=gcol[ggroup$hc$labels],
        RowSideColors=gcol[ggroup$hc$labels],
        col=colorRampPalette(c('black', 'white'))(100)
)
```

We can also visualize these spatially clustered genes within their identified spatial patterns as a heatmap. Again, each column is a voxel ordered and colored by their identified cell-type from our clustering analysis. Each row is a significantly spatially clustered gene ordered and colored by their identified spatial pattern.

```
# Plot as heatmap
sp.genes <- unlist(lapply(levels(ggroup$groups), function(x) {
  names(ggroup$groups)[ggroup$groups==x]
}))
ccol <- rainbow(length(levels(annot)))[annot]
names(ccol) <- names(annot)

m <- as.matrix(mat[sp.genes,names(sort(annot))])
m <- winsorize(t(scale(t(m))))
heatmap(m, scale="none", 
          Colv=NA, Rowv=NA, labRow=NA, labCol=NA,
          ColSideColors=ccol[colnames(m)],
          RowSideColors=gcol[rownames(m)],
          col=colorRampPalette(c('blue', 'white', 'red'))(100)
)
```

In this particular case, as cell-types in the mOB are inherently spatially organized, we see a strong correspondence between spatially clustered genes and the cell-type markers we identified previously. We can assess the significance of the overlaps between our spatially-aggregated gene sets and our previous differentially upregulated gene sets using a hypergeometric test. Indeed, patterns appears to mark combinations of spatially-colocalized cell-types.

```
# Compare two different types of identifying genes
diffgexp <- dg.sig
spatgexp <- lapply(levels(ggroup$groups), function(x) {
  names(ggroup$groups)[ggroup$groups==x]
})
names(spatgexp) <- paste0('Spatial Pattern ', levels(ggroup$groups))

# Assess significance of overlap by hypergeometric test
sigoverlap <- do.call(rbind, lapply(1:length(spatgexp), function(i) {
  so <- unlist(lapply(1:length(diffgexp), function(j) {
    
    #x = # of genes in common between two groups.
    #n = # of genes in group 1.
    #D = # of genes in group 2.
    #N = total genes
    x <- length(intersect(spatgexp[[i]], diffgexp[[j]])) ## shared
    n <- length(spatgexp[[i]])
    D <- length(diffgexp[[j]])
    N <- nrow(counts) ## total
    
    phyper(x, D, N-D, n, lower.tail=FALSE)
  }))
  names(so) <- names(diffgexp)
  return(so)
}))
rownames(sigoverlap) <- 1:length(spatgexp)

# Visualize as heatmap
pvo <- sigoverlap
rownames(pvo) <- paste0('spatial: ', rownames(sigoverlap))
colnames(pvo) <- colnames(sigoverlap)
pvo[pvo < 1e-6] <- 1e-6 # prevent Infs
pvo <- -log10(pvo)
# order for diagonal
matrix.sort <- function(matrix) {
  row.max <- apply(matrix,1,which.max)
  if(all(table(row.max) != 1)) stop("Ties cannot be resolved")
  matrix[names(sort(row.max)),]
}
pvo <- matrix.sort(pvo)
heatmap(pvo, 
        col=colorRampPalette(c('white', 'black'))(100), 
        scale="none", Rowv=NA, Colv=NA, margins = c(25,15))
```

We can also visualize the expression of a few genes in space. We will focus on genes upregulated in the Granule Cell Layer and significantly spatially aggregated in Spatial Pattern 1. All of these genes have been identified as significantly spatially aggregated. We will use a signed Local Indicators of Spatial Autocorrelation (sLISA) plot to show which probe spots are driving this global spatial aggregation.

```
# look at genes 
gs <- intersect(spatgexp[["Spatial Pattern 2"]], dg.sig[['1: Granular Cell Layer']])
# order by degree of spatial clustering
gs <- gs[order(I[gs,]$observed, decreasing=TRUE)]

# plot
par(mfrow=c(2,2), mar=rep(2,4))
invisible(lapply(gs[1:2], function(g) {
  gexp <- winsorize(scale(mat[g,])[,1])
  plotEmbedding(pos, colors=gexp, cex=2, main=g, verbose=FALSE)
  slisa <- winsorize(signedLisa(gexp, w))
  plotEmbedding(pos, colors=slisa, gradientPalette=colorRampPalette(c('darkgreen', 'white', 'darkorange'))(100), cex=2)
}))
```

# Complementary analysis: Intra-cell-type spatial heterogeneity

Spatial analysis can also be complementary to spatially-unaware clustering analysis. For example, after identifying putative cell-types by spatially-unaware clustering analysis, we may be interesting in identifying genes that exhibit spatial clustering within this cell-type. For demonstration purposes, we will focus on testing whether any marker genes for the Granule Cell Layer exhibit spatial clustering within the Granular Cell Layer.

```
# Restrict to just voxels corresponding to the Granular Cell Layer.
sub <- names(annot)[annot == '1: Granule Cell Layer']
w.sub <- getSpatialNeighbors(pos[sub,], filterDist = 2.5)
plotNetwork(pos[sub,], w.sub)
```

```
gs <- dg.sig[['1: Granule Cell Layer']]
I.sub <- getSpatialPatterns(mat[gs, sub], w.sub)
results.filter.sub <- filterSpatialPatterns(mat = mat[, sub],
                                        I = I.sub,
                                        w = w.sub,
                                        adjustPv = TRUE,
                                        alpha = 0.05,
                                        minPercentCells = 0.05,
                                        verbose = TRUE)
```

```
## Number of significantly autocorrelated genes: 0
```

```
## ...driven by > 3.3 cells: 0
```

In this case, we do not identify any additional aspects of spatial heterogeneity within this cell-type.

# Additional exercises

1. Are there additional aspects of spatial heterogeneity within other cell layers?
2. Are there any shared aspects of spatial heterogeneity, potentially indicative of shared spatial gradients, shared across cell-types?
3. The mOB exhibits bilateral symmetry. Are the spatially aggregated genes identified in the left lobe consistent with the spatially aggregated genes identified in the right lobe?
