# Supplementary material for: Characterizing spatial gene expression heterogeneity in spatially resolved single-cell transcriptomic data with nonuniform cellular densities
Source: Genome Res. 2021 Oct;31(10):1843–55. doi: 10.1101/gr.271288.120 (PMC8494224; doi:10.1101/gr.271288.120)
Supplement: Supplemental Material [file supp_gr.271288.120_Supplemental_Software_MERINGUE_1.0.tar.gz › MERINGUE/inst/doc/mouseCoronal.html]

Spatial Transcriptomics Analysis of the Mouse Brain with MERINGUE


# Spatial Transcriptomics Analysis of the Mouse Brain with MERINGUE

#### Brendan F. Miller and Jean Fan

In this vignette, we will walk through an analysis of spatial transcriptomics (ST) data for a coronal section of the mouse brain. The mouse brain data for 2702 ST “spots” has been prepared for you and is available as a part of the package. Here, `tissueSpotRotation` is a dataframe where each row is a probe spot’s x and y positions in space, and `filteredGenes` is a CPM normalized and log-transformed gene expression matrix with a filtered set of 1263 genes whose expression variance across the 2702 spots is higher than transcriptome-wide expectations.

```
library(MERINGUE)
library(Matrix)

data("mouseCoronal")
filteredGenes <- mouseCoronal$filteredGenes
tissueSpotRotation <- mouseCoronal$tissueSpotRotation
```

First, let’s cluster the spots into transcriptionally distinct cell-types and subtypes. We will use spatially-unaware dimensionality reduction just based on gene expression without explicit consideration for spatial information. G=However, given the known spatial organization of cell-types in the brain, we anticipate that identified clusters should correspond to distinct cell layers or other spatially organized structures.

```
# Dimensionality reduction by PCA on log10 CPM normalized expression values
pcs.info <- prcomp(t(log10(as.matrix(filteredGenes)+1)), center=TRUE)
# Check `screeplot` to assess number of PCs to use
screeplot(pcs.info, npcs=20,)
```

```
# Choose number of PCs to look at based on screeplot ("elbow rule")
numberPcs <- 5 #
pcs <- pcs.info$x[,1:numberPcs]
# 2D embedding by tSNE
emb <- Rtsne::Rtsne(pcs,
             is_distance=FALSE,
             perplexity=30,
             num_threads=1,
             verbose=FALSE)$Y
rownames(emb) <- rownames(pcs)
# iGraph cluster community labeling
spotClusters <- getClusters(pcs, k = 100)
```

This approach identified 8 transcriptionally distinct clusters of cells. Indeed, each of the 8 clusters appear to correspond to a particular cell layer or region in the mouse coronal brain slice.

```
par(mfrow=c(1,2), mar=rep(2,4))
# plot the tsne and color by community
plotEmbedding(emb, groups=spotClusters, 
              show.legend=TRUE, xlab='tSNE X', ylab='tSNE Y',
              verbose=FALSE)
              
# plot the spots based on position on tissue, color by community
plotEmbedding(tissueSpotRotation, groups=spotClusters, 
              cex=1, xlab='spatial X', ylab='spatial Y',
              verbose=FALSE)
```

Interestingly, cluster 4 traces the hippocampus proper CA1, CA2, and CA3 feilds quite well, but there are other spots of this cluster present in potentially the olfactory areas of the cerebral cortex. It is possible that this cluster corresponds to pyramidal cells. To further interpret each cluster 4, let’s identify some marker genes that are significantly enriched.

```
# Identify significantly differentially upregulated genes
# in each identified cluster by Wilcox test
diffGenes <- getDifferentialGenes(as.matrix(filteredGenes), spotClusters)
## focus on cluster 4
diffExpCluster4Genes <- diffGenes[[4]]
# gene with highest expression in cluster assigned as marker to that cluster
highestExpClust4 <- diffExpCluster4Genes[which(diffExpCluster4Genes$highest == TRUE),]
## order and view
topDiffExpGenesClust4 <- highestExpClust4[order(highestExpClust4$p.adj),]
topDiffExpGenesClust4[1:10,]
```

```
## [1] "Running differential expression with 8 clusters ... "
## [1] "Summarizing results ... "
##                p.adj        Z           M highest        fe
## Cpne6   1.187723e-68 17.47118 -0.05263544    TRUE 1.0000000
## Wasf1   1.050439e-67 17.34638 -0.38096373    TRUE 1.0000000
## Fam131a 3.843030e-65 17.00388 -0.17434450    TRUE 1.0000000
## Wipf3   1.837057e-64 16.91194  0.36127640    TRUE 0.9920635
## Icam5   5.984617e-63 16.70542 -0.03396774    TRUE 1.0000000
## Plppr2  5.164388e-62 16.57637 -0.32489919    TRUE 1.0000000
## Nell2   1.259865e-59 16.24261 -0.41680245    TRUE 1.0000000
## Scn3b   5.197973e-55 15.57723  0.04819406    TRUE 1.0000000
## Limd2   5.431856e-55 15.57442 -0.11120479    TRUE 1.0000000
## Mmd     4.541597e-54 15.43803 -0.38680633    TRUE 1.0000000
```

Looking at ISH data, several of these genes with coronal brain slice ISH data trace the hippocampus proper pyrimidal cell layer well and have expression in the olifactory region layer that was included in cluster 4. The top hit, Cpne6, has been reported to be expressed in pyramidal cells of the CA1-3 regions of the hippocampus.

```
par(mfrow=c(1,2), mar=rep(2,4))
g <- 'Cpne6'
gexp <- scale(filteredGenes[g,])[,1]
plotEmbedding(emb, col=gexp)
plotEmbedding(tissueSpotRotation, col=gexp, 
              cex=1, xlab='spatial X', ylab='spatial Y')
```

So transcriptionally distinct cluster 4 appears to have identified pyramidal neurons of the hippocampus proper and potentially a pyramidal layer in the olfactory region. The hippocampus proper is a highly specific and striking structure of the brain. During development, one hypothesis is that gene expression gradients are responsible for the organization of cell types into their respective positions and thus drive the formation of tissues and cellular structures. We can further analyze these putative pyramidal cells to identify such potential additional spatial gene expression gradients.

We will first build an adjacency matrix of spots in cluster 4.

```
# get spots that are part of the cluster
cluster4SpotIDs <- names(spotClusters[which(spotClusters == '4')])
cluster4SpotCoords <- tissueSpotRotation[cluster4SpotIDs,]
# build weight matrix for the cluster spots
cluster4WeightMatrix <- getSpatialNeighbors(
  cluster4SpotCoords, filterDist = 25, verbose=TRUE)
```

```
## Filtering by distance: 25...
```

```
## Binarizing adjacency weight matrix ...
```

```
## Done!
```

```
plotNetwork(cluster4SpotCoords, cluster4WeightMatrix)
```

We can then identify significantly spatially variable genes.

```
# get gene expression of filteredGenes for just the cluster spots
cluster4GeneExp <- filteredGenes[,which(colnames(filteredGenes) %in% cluster4SpotIDs)]

# calculate Moran's I for filteredGenes only using the spots in cluster
# aka autocorrelation of filtered genes within the cluster
cluster4GeneExpMoransI <- getSpatialPatterns(cluster4GeneExp,
                                           cluster4WeightMatrix,
                                           verbose=TRUE)

# find significantly spatially variable genes
cluster4SpatialGenes <- filterSpatialPatterns(
  cluster4GeneExp,
  cluster4GeneExpMoransI,
  cluster4WeightMatrix,
  details = TRUE,
  verbose=TRUE,
  minPercentCells = 0.10)
```

```
## Number of significantly autocorrelated genes: 737
```

```
## ...driven by > 12.6 cells: 651
```

```
# plot a few genes
par(mfrow=c(2,2))
sapply(rownames(cluster4SpatialGenes)[1:4], function(g) {
  plotEmbedding(emb = cluster4SpotCoords,
                col = winsorize(scale(cluster4GeneExp[g,])[,1]),
                main=g)
})
```

```
## $Vxn
## NULL
## 
## $Ogfrl1
## NULL
## 
## $`2010300C02Rik`
## NULL
## 
## $Pantr1
## NULL
```

Indeed, we are able to identify a few hundred significantly spatially variable genes. Let’s try to organize them into spatial patterns.

```
# spatial cross cor matrix
cluster4CorrMtx <- spatialCrossCorMatrix(
  cluster4GeneExp[rownames(cluster4SpatialGenes),],
  cluster4WeightMatrix)

par(mfrow=c(2,2))
# use all tissue spots to make the visualization more representative
method = 'ward.D'
cluster4SpatialPatterns <- groupSigSpatialPatterns(
  pos=tissueSpotRotation,
  mat=cluster4GeneExp[rownames(cluster4SpatialGenes),],
  scc=cluster4CorrMtx,
  hclustMethod = method,
  deepSplit = 1,
  binSize = 50,
  power = 1)
```

```
## Patterns detected:
```

```
# Double check cross correlation matrix
par(mfrow=c(1,1))
# Look at pattern association by plotting SCI matrix as a heatmap and dendrogram
patternColors <- rainbow(
  length(levels(cluster4SpatialPatterns$groups)), v=0.5)[cluster4SpatialPatterns$groups]
names(patternColors) <- names(cluster4SpatialPatterns$groups)
# Visualize as heatmap
heatmap(cluster4CorrMtx[cluster4SpatialPatterns$hc$labels, cluster4SpatialPatterns$hc$labels],
        scale='none', 
        Colv=as.dendrogram(cluster4SpatialPatterns$hc), 
        Rowv=as.dendrogram(cluster4SpatialPatterns$hc), 
        labCol=NA, labRow = NA,
        RowSideColors=patternColors[cluster4SpatialPatterns$hc$labels],
        ColSideColors=patternColors[cluster4SpatialPatterns$hc$labels],
        col=colorRampPalette(c('white', 'lightgrey', 'black'))(100)
)
```

```
##  ..cutHeight not given, setting it to 80.7  ===>  99% of the (truncated) height range in dendro.
##  ..done.
## groups
##   1   2   3   4 
## 238 183 148  82 
## [1] "Filling ..."
## [1] "Filling ..."
## [1] "Filling ..."
## [1] "Filling ..."
```

Interestingly, the spatial patterns appear to distinguish the putative pyramidal neuron layer in the olfactory area (spatial pattern 1), and also appear to demarcate the CA1 (spatial pattern 3), CA2 (spatial pattern 4), and CA3 (spatial pattern 2) regions of the hippocampus proper.

# Additional exercises

1. Are there additional aspects of spatial heterogeneity within other cell layers?
2. Do these aspects of spatial heterogeneity correspond to known anatomical structures?
3. Are these patterns of spatial heterogeneity within cell layers shared across different brain regions?
