# Supplementary material for: Characterizing spatial gene expression heterogeneity in spatially resolved single-cell transcriptomic data with nonuniform cellular densities
Source: Genome Res. 2021 Oct;31(10):1843–55. doi: 10.1101/gr.271288.120 (PMC8494224; doi:10.1101/gr.271288.120)
Supplement: Supplemental Material [file supp_gr.271288.120_Supplemental_Software_MERINGUE_1.0.tar.gz › MERINGUE/inst/doc/simulation.html]

Understanding MERINGUE’s Spatial Cross-Correlation Statistic using Simulations


# Understanding MERINGUE’s Spatial Cross-Correlation Statistic using Simulations

#### Jean Fan

#### February 14, 2019

In order to cluster genes that mark similar spatial patterns in space as well as infer evidence of cellular communication between spatially co-localized cell-types, MERINGUE computes a spatial cross-correlation statistic. In this tutorial, we will explore the distinction between this spatial cross-correlation statistic compared to a general (spatially-unaware) cross-correlation using simulations.

```
suppressMessages(library(MERINGUE))
```

# Simulate cells in space

First, let’s simulate some cells in space. Each point here is a cell. Their location in the plot can be interpreted as their physical location in space.

```
# 15x15 grid of cells
N <- 15^2
pos <- t(combn(c(1:sqrt(N), rev(1:sqrt(N))), 2))
pos <- unique(pos)
rownames(pos) <- paste0('cell', 1:N)
colnames(pos) <- c('x', 'y')
# jitter
posj <- jitter(pos, amount = 0.5)
# induce warping
posw <- 1.1^posj
# plot
par(mfrow=c(1,1), mar=rep(5,4))
plotEmbedding(posw,  main='Simulated Cells in Space')
```

Next, let’s simulate various gene expression patterns to highlight different scenarios that will help highlight the distinction spatial cross-correlation versus general (spatially-unaware) cross-correlation.

# Scenario 1: General cross-correlation and spatial cross-correlation suggest similar trends

First, let’s consider two genes, `Gene1` and `Gene2`. Both genes are expressed in all cells but along a gradient. Cells spatially located towards the left will generally have higher expression of `Gene1` and also higher expression of `Gene2` compared to cells on the right. We can visualize these gradients by coloring cells based on their expression levels of the two genes.

```
par(mfrow=c(1,2), mar=rep(5,4))
gexp0 <- sort(abs(rnorm(N)))

set.seed(0)
gexp1 <- jitter(gexp0, amount = 0.5)
names(gexp1) <- rownames(pos)
plotEmbedding(posw, col=gexp1,  
              main='Gene1')

set.seed(1)
gexp2 <- jitter(gexp0, amount = 0.5)
names(gexp2) <- rownames(pos)
plotEmbedding(posw, col=gexp2,  
              main='Gene2')
```

If we plot the expression of `Gene1` versus `Gene2`, as expected, we see a positive relationship. Likewise, if we compute a general cross-correlation statistic between `Gene1` and `Gene2`, we can identify a significant positive cross-correlation - that is, cells that express higher levels of `Gene1` tend to express higher levels of `Gene2` and cells that express lower levels of `Gene1` tend to express lower levels of `Gene2`.

```
# Plot
par(mfrow=c(1,1), mar=rep(5,4))
plot(gexp1, gexp2, 
     main='Scatterplot of\nGene1 versus Gene2')
abline(lm(gexp2~gexp1))
```

```
# Compute cross correlation
cor.test(gexp1, gexp2)
```

```
## 
##  Pearson's product-moment correlation
## 
## data:  gexp1 and gexp2
## t = 22.786, df = 223, p-value < 2.2e-16
## alternative hypothesis: true correlation is not equal to 0
## 95 percent confidence interval:
##  0.7922577 0.8718055
## sample estimates:
##       cor 
## 0.8363825
```

Likewise, if we compute a spatial cross-correlation statistic between `Gene1` and `Gene2`, we can identify a significant positive spatial cross-correlation - that is, cells that express higher levels of `Gene1` tend to be spatially neighboring cells that tend to express higher levels of `Gene2` and cells that express lower levels of `Gene1` tend to be spatially neighboring cells that tend to express lower levels of `Gene2`.

```
weight <- getSpatialNeighbors(posw, filterDist = 1)
plotNetwork(posw, weight)
```

```
spatialCrossCor(gexp1, gexp2, weight)
```

```
## [1] 0.8105344
```

```
par(mfrow=c(1,1), mar=rep(5,4))
spatialCrossCorTest(gexp1, gexp2, weight, 
                    plot=TRUE)
```

```
## [1] 0.000999001
```

In this case, both the general and spatial cross-correlation statistics are positive.

# Scenario 2: General cross-correlation and spatial cross-correlation suggest different trends

Now, let’s consider different two genes, `Gene3` and `Gene4`. `Gene3` is expressed in a subset of cells along a gradient. `Gene4` is expressed in a different subset of cells, but along a similar gradient.

```
par(mfrow=c(1,2), mar=rep(5,4))
num <- pos[,1]

vi <- (num %% 2) == 0
gexp3 <- as.numeric(vi)
gexp3 <- gexp3 * sort(abs(rnorm(length(gexp3)))+1)
names(gexp3) <- rownames(pos)
plotEmbedding(posw, col=gexp3,  
              main='Gene3')

vi <- (num %% 2) == 1
gexp4 <- as.numeric(vi)
gexp4 <- gexp4 * sort(abs(rnorm(length(gexp4)))+1)
names(gexp4) <- rownames(pos)
plotEmbedding(posw, col=gexp4,  
              main='Gene4')
```

Now, if we plot the expression of `Gene3` versus `Gene4` in a scatterplot, we see a negative relationship between the two genes are expressed in different subsets of cells. Likewise, if we compute a general cross-correlation statistic between `Gene3` and `Gene4`, we can identify a significant negative cross-correlation - that is, cells that express higher levels of `Gene3` tend to express lower levels of `Gene4` and cells that express higher levels of `Gene4` tend to express lower levels of `Gene3`.

```
# Plot
par(mfrow=c(1,1), mar=rep(5,4))
plot(gexp3, gexp4, 
     main='Scatterplot of\nGene3 versus Gene4')
abline(lm(gexp4~gexp3))
```

```
# Compute cross correlation
cor.test(gexp3, gexp4)
```

```
## 
##  Pearson's product-moment correlation
## 
## data:  gexp3 and gexp4
## t = -21.655, df = 223, p-value < 2.2e-16
## alternative hypothesis: true correlation is not equal to 0
## 95 percent confidence interval:
##  -0.8612940 -0.7760043
## sample estimates:
##        cor 
## -0.8232408
```

However, if we compute a spatial cross-correlation statistic between `Gene3` and `Gene4`, we can identify a significant positive spatial cross-correlation - that is, cells that express higher levels of `Gene3` tend to be spatially neighboring cells that tend to express higher levels of `Gene4` and cells that express lower levels of `Gene3` tend to be spatially neighboring cells that tend to express lower levels of `Gene4`.

```
# Compute spatial cross correlation
spatialCrossCor(gexp3, gexp4, weight)
```

```
## [1] 0.1463269
```

```
par(mfrow=c(1,1), mar=rep(5,4))
spatialCrossCorTest(gexp3, gexp4, weight, 
                    plot=TRUE)
```

```
## [1] 0.000999001
```

In this case, even though the general cross-correlation statistic is negative, the spatial cross-correlation statistic is positive.

This distinction is particularly important when we consider how transcriptionally-distinct cell-types and subtypes may be interacting with each other in space. For example, consider if `Gene3` is a receptor and `Gene4` is a ligand. A general (spatially-unaware) cross-correlation would not point us to any relationship between `Gene3` and `Gene4` other than that they are expressed on different cell-types or subtypes. But a spatial (spatially-aware) cross-correlation would hint at an interaction.

## Computing an inter-cell-type spatial cross-correlation

Now, let’s call cells that expression `Gene3` cell-type A. And let’s call cells that express `Gene4` cell-type B. Note that cells of cell-type A and cells of cell-type by are spatially intertwined.

```
ctA <- names(gexp3)[gexp3>0]
ctB <- names(gexp4)[gexp4>0]

# double check mutually exclusive
print(intersect(ctA, ctB))
```

```
## character(0)
```

```
# plot
par(mfrow=c(1,1), mar=rep(5,4))
cellType <- factor(rownames(posw) %in% ctA)
levels(cellType) <- c('ctB', 'ctA')
names(cellType) <- rownames(posw)
plotEmbedding(posw, groups=cellType, 
              show.legend=TRUE,  
              main='Cell Types in Space')
```

Now, instead of considering all neighbors, because we can see two transcriptionally distinct but spatially intertwined cell-types in our data, let’s only consider neighbor-relationships between cells of cell-type A and cells of cell-type B. We can acheive this by modifying the binary weight matrix used in the spatial cross-correlation statistic calculation to include only neighbor-relationships between the two cell-types (as opposed to within each cell-type). And indeed, we see a very high inter-cell-type spatial cross-correlation - that is, cells of cell-type A that express higher levels of `Gene3` tend to be spatially neighboring cells of cell-type B that tend to express higher levels of `Gene4` and cells of cell-type A that express lower levels of `Gene3` tend to be spatially neighboring cells of cell-type B that tend to express lower levels of `Gene4`, and vice versa.

```
par(mfrow=c(1,1), mar=rep(5,4))
weightIc <- getInterCellTypeWeight(ctA, ctB, 
                                   weight, posw, 
                                   plot=TRUE, 
                                   main='Adjacency Weight Matrix\nBetween Cell-Types')
```

```
spatialCrossCor(gexp3, gexp4, weightIc)
```

```
## [1] 0.9576565
```

```
interCellTypeSpatialCrossCor(gexp3, gexp4, ctA, ctB, weightIc)
```

```
## [1] -0.1197252
```

```
spatialCrossCorTest(gexp3, gexp4, weightIc)
```

```
## [1] 0.000999001
```

```
plotInterCellTypeSpatialCrossCor(gexp3, gexp4, ctA, ctB, weightIc,
                                 main='Spatial Cross Correlation')
```

Indeed, if `Gene3` is a receptor and `Gene4` is a ligand, the observation that higher expression of the receptor in one cell-type is always to spatially co-localized with higher expression of the ligand in a different cell-type could be indicative of cellular interactions between cell-Type A and B via these receptor-ligand complexes.

# Scenario 3: Neither general cross-correlation nor spatial cross-correlation

Lastly, let’s consider two genes, `Gene5` and `Gene6`. `Gene5` exhibits a spatial gradient going from left to right. And `Gene6` exhibits a spatial gradient going from top to down.

```
par(mfrow=c(1,2), mar=rep(2,4))
set.seed(0)
gexp5 <- jitter(pos[,1], amount = 0.5)
names(gexp5) <- rownames(pos)
plotEmbedding(posw, col=gexp5,  
              main='Gene5')

set.seed(1)
gexp6 <- jitter(pos[,2], amount = 0.5)
names(gexp6) <- rownames(pos)
plotEmbedding(posw, col=gexp6,  
              main='Gene6')
```

We observe no significant cross-correlation relationships between the two genes.

```
# Plot
par(mfrow=c(1,1), mar=rep(2,4))
plot(gexp5, gexp6, main='Scatterplot of\nGene5 versus Gene6')
abline(lm(gexp5~gexp6))
```

```
# Compute cross correlation
cor.test(gexp5, gexp6)
```

```
## 
##  Pearson's product-moment correlation
## 
## data:  gexp5 and gexp6
## t = 0.079181, df = 223, p-value = 0.937
## alternative hypothesis: true correlation is not equal to 0
## 95 percent confidence interval:
##  -0.1255754  0.1359986
## sample estimates:
##         cor 
## 0.005302307
```

And also no significant spatial cross-correlation in this case.

```
# Compute spatial cross correlation
spatialCrossCor(gexp5, gexp6, weight)
```

```
## [1] 0.003494172
```

```
par(mfrow=c(1,1), mar=rep(2,4))
spatialCrossCorTest(gexp5, gexp6, weight, 
                    plot=TRUE)
```

```
## [1] 0.965035
```

Despite neither gene showing any spatial or general cross-correlation relationship between them, both genes can and do still exhibit high spatial auto-correlation in this example.

In summary, as these various simulated gene expression patterns highlight, spatial cross-correlation and autocorrelation can provide complementary information to general correlation analyses to enable the identification of potentially interesting spatial patterns indicative of cellular communication.
