# Supplementary material for: Characterizing spatial gene expression heterogeneity in spatially resolved single-cell transcriptomic data with nonuniform cellular densities
Source: Genome Res. 2021 Oct;31(10):1843–55. doi: 10.1101/gr.271288.120 (PMC8494224; doi:10.1101/gr.271288.120)
Supplement: Supplemental Material [file supp_gr.271288.120_Supplemental_Software_MERINGUE_1.0.tar.gz › MERINGUE/inst/doc/spatial_clustering.html]

Spatially-informed transcriptional clustering with MERINGUE


# Spatially-informed transcriptional clustering with MERINGUE

#### Jean Fan

# Introduction

The spatial organization and positioning of cells play important roles in shaping cellular identities. In a variety of biological systems (from the fly embryo to the mammalian brain), we have seen how such spatial distinct organization is reflected in the cellular transcriptome. Thus, we generally expect unsupervised clustering based on transcriptional profiles alone to recover spatially organized cell populations. However, what if we take into consideration the spatial positioning in additional to transcriptional profiles in our identication of cellular clusters? Would this allow us to resolve more subtly transcriptionally different but spatially distinct cellular subpopulations? In this vignette, we will explore the utility of this spatially-informed transcriptional clustering using simulated examples.

# Simulation

First, let’s simulate 3 spatially distinct groups of cells. Each group will have 300 cells that are spatially positioned in distinct groups. We will further color these cells by their groups.

```
library(MERINGUE)
par(mfrow=c(1,1), mar=rep(4,4))

## simulate 3 spatially but 2 transcriptionally distinct groups
N <- 300
## Three spatially distinct groups
pos1 <- cbind(rnorm(N/3), rnorm(N/3))
pos2 <- cbind(rnorm(N/3, 10), rnorm(N/3))
pos3 <- cbind(rnorm(N/3, 10), rnorm(N/3, 10))
pos <- rbind(rbind(pos1, pos2), pos3)
group <- c(rep(1, N/3), rep(2, N/3), rep(3, N/3))
names(group) <- rownames(pos) <- paste0('cell', 1:N)
plotEmbedding(pos, groups=group, main='Cell-Types in Space', xlab='Spatial X', ylab='Spatial Y')
```

Now, let’s simulate a situation where two of these groups of cells are transcriptionally identical. We will have 30 genes that are upregulated in the blue group of cells compared to the green and red cells and 60 genes that are upregulated in the green and red cells relative to the blue cells. But there are no significant transcriptional differences between green and red cells.

```
M <- 30
## But two are transcriptionally identical
pcs12 <- matrix(rnorm(N*2/3*M), N*2/3, M)
pcs3 <- matrix(rnorm(N*1/3*M, 10), N*1/3, M)
pcs <- rbind(pcs12, pcs3)
pcs <- cbind(pcs, abs(10-pcs))
colnames(pcs) <- paste0('gene', 1:ncol(pcs))
rownames(pcs) <- rownames(pos)
heatmap(pcs, scale="none", Rowv = NA, Colv=NA,
        RowSideColors=MERINGUE:::fac2col(group),
        col=colorRampPalette(c('blue', 'white', 'red'))(100))
```

Indeed, when we visualize the expression of these genes in space, we can see that they mark either only the cells in the blue group or cells in both the red and green groups.

```
par(mfrow=c(1,2))
plotEmbedding(pos, colors=scale(pcs[,1])[,1],
              main='Cell-Types Gene Expression', xlab='Spatial X', ylab='Spatial Y')
plotEmbedding(pos, colors=scale(pcs[,50])[,1],
              main='Cell-Types Gene Expression', xlab='Spatial X', ylab='Spatial Y')
```

So what happens when we perform transcriptome-based dimensionality reduction with UMAP? Expectedly, we identify two transcriptionally distinct clusters of cells.

```
######### See if we can integrate spatial information in cluster detection
par(mfrow=c(1,2))
emb <- uwot::umap(pcs, min_dist = 0.5)
rownames(emb) <- rownames(pcs)
plotEmbedding(emb, col=scale(pcs[,1])[,1], 
              main='Cell-Types Gene Expression', xlab='UMAP X', ylab='UMAP Y')
plotEmbedding(emb, col=scale(pcs[,50])[,1],
              main='Cell-Types Gene Expression', xlab='UMAP X', ylab='UMAP Y')
```

Likewise, when we perform transcriptional-based clustering, we identify the two transcriptionally distinct clusters of cells.

```
## First, with standard spatially-unaware cluster detection
par(mfrow=c(1,2))
com <- getClusters(pcs, k=50)
plotEmbedding(pos, groups=com, main='Transcriptional Clusters', xlab='Spatial X', ylab='Spatial Y')
plotEmbedding(emb, groups=com, main='Transcriptional Clusters', xlab='UMAP X', ylab='UMAP Y')
```

However, given that two of these groups of cells are spatially distinct, perhaps we would like to integrate the spatial information in order to tease them apart. Briefly, as in expression-based clustering, we first construct a neighbor graph where nodes are cells and nodes are connected with an edge if the represented cells that are within the k-most transcriptionally similar cells. To integrate spatial information, we then weigh the edges of this transcriptionally defined graph by the density agnostic distance between two cells in the adjacency representation W. In this manner, cells that are transcriptionally similar AND also spatially proximal in space will be given greater consideration in downstream graph-based community detection for clustering. We find such a graph-based weighting approach to be more stable than directly incorporating spatial positioning information in defining the distance between cells in the initial neighbor graph construction.

```
W <- getSpatialNeighbors(pos, filterDist = 2)
plotNetwork(pos, W)
```

```
com2 <- getSpatiallyInformedClusters(pcs, W=W, k=50)
table(com2)
plotEmbedding(pos, groups=com2, main='Spatially Aware Transcriptional Clusters', xlab='Spatial X', ylab='Spatial Y')
```

```
plotEmbedding(emb, groups=com2, main='Spatially Aware Transcriptional Clusters', xlab='UMAP X', ylab='UMAP Y')
```

```
## com2
##   1   2 
## 200 100
```

We thus show how incorporation of spatial information may help us resolve spatially distinct but transcriptionally similar cellular subpopulations. In real biology however, bi-lateral symmetry and other organismal symmetries may result in transcriptionally homogeneous but spatially distinct organization of the same cell-type. In such a case, we may not want to consider these as different subpopulations. Indeed, there are no statistically significant differentially expressed genes between our two identified transcriptionally homogeneous but spatially distinct groups of cells.

```
## what's different between our spatially aware and unaware clustering
print(table(com, com2))
## try to identify subtly differentially expressed genes
vi <- com2 %in% c(1,2)
cols <- factor(com2[vi])
diffgexp <- getDifferentialGenes(t(pcs[vi,]), cols)
sigdiffgexp <- sapply(diffgexp, function(x) sum(x$p.adj < 0.05))
sigdiffgexp
```

```
##    com2
## com   1   2
##   1 200   0
##   2   0 100
## [1] "Running differential expression with 2 clusters ... "
## [1] "Summarizing results ... "
##  1  2 
## 30 30
```

We thus suggest that such spatially informed clustering to be complementary to differential expression analysis, whereby identified clusters of spatially distinct cells should be analyzed for significantly differentially expressed genes to ensure the presence of significant, likely subtle, transcriptional differences.
